# Synthesis and Evaluation of New Podophyllotoxin Derivatives with *in Vitro* Anticancer Activity

**DOI:** 10.3390/molecules200712266

**Published:** 2015-07-06

**Authors:** Wei-Hua Cheng, Hai Shang, Cong Niu, Zhong-Heng Zhang, Li-Ming Zhang, Hong Chen, Zhong-Mei Zou

**Affiliations:** 1 Institute of Medicinal Plant Development, Chinese Academy of Medical Sciences and Peking Union Medical College, Beijing 100193, China; E-Mails: cheng1083@163.com (W.-H.C.); shanghai0503@163.com (H.S.); 2 Pharmacognosy Division, Medical College of Chinese People’s Armed Police Force, Tianjin 300162, China; E-Mail: anthritynika@163.com; 3 Tianjin Key Laboratory on Technologies Enabling Development of Clinical Therapeutics and Diagnostics, School of Pharmacy, Tianjin Medical University, Tianjin 300070, China; E-Mails: zhanghengxb@163.com (Z.-H.Z.); 13920122174@163.com (L.-M.Z.)

**Keywords:** podophyllotoxin, MDR, synthesized, antitumor activity

## Abstract

A series of novel podophyllotoxin derivatives were designed and synthesized. The cytotoxic activities of these compounds were tested against three tumor cell lines (HeLa, K562, and K562/A02). Most of the derivatives (IC_50_ = 1–20 μM) were found to have stronger cell growth inhibitory activity than positive control etoposide. Among them, 4β*-N*-[(*E*)-(5-((4-(4-nitrophenyl)-piperazin-1-yl)methyl)furan-2-yl)prop-2-en-1-amine]-4-desoxy-podophyllotoxin (9l) demonstrated significant inhibitory activity against HeLa, K562, and K562/A02 cell lines with IC_50_ values of 7.93, 6.42, 6.89 μM, respectively.

## 1. Introduction

Cancer is a major public health problem in the world. In 2008, 7.6 million people died of cancer (around 13% of all deaths), and this number is projected to increase with an estimated 13.1 million in 2030 [1].

**Figure 1:**
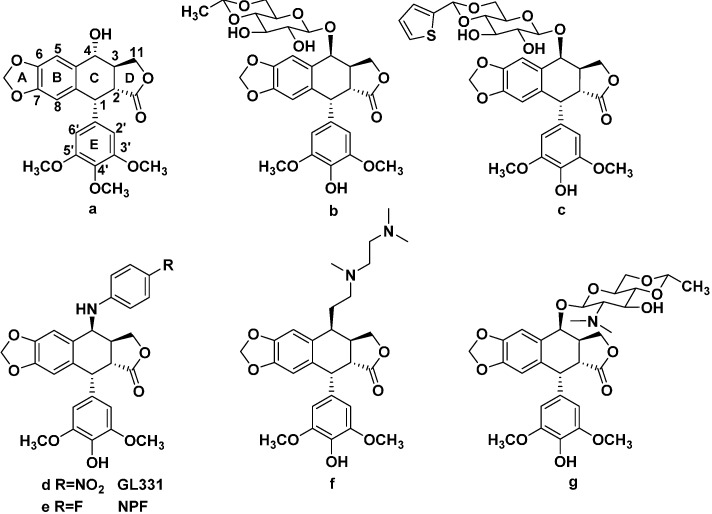
Structures of podophyllotoxin (**a**); etoposide (**b**); teniposide (**c**); GL-331 (**d**); NPF (**e**); TOP-53 (**f**); NK-611 (**g**).

Podophyllotoxin (PPT, **a**), the most abundant naturally occurring cyclolignan isolated mainly from *Podophyllum peltatum* and *P. hexandrum*, has important antineoplastic and antiviral properties [2]. However, its antimitotic activity is proved to be of the greatest interest to researchers [3]. Because of its toxic side effects, extensive structural modifications were performed since the 1950s. Podophyllotoxin derivatives possess antitumor activity, such as etoposide (VP-16, **b**) and teniposide (VM-26, **c**) (Figure 1) have been widely used as anticancer drugs for clinical chemotherapy [4]. However, their low water solubility, acquired drug-resistance and severe gastrointestinal disturbances have promoted the search for new derivatives of podophyllotoxin [5]. The structural modifications and mechanism of action of podophyllotoxin have been studied over the years and the C4 position is considered potentially the most modifiable position. Diverse analogs like GL-331 (**d**), NPF (**e**), TOP-53 (**f**), NK-611 (**g**) (Figure 1), which are presently under clinical trial have been developed [6,7,8].

Investigation of the structure-activity relationships of PPT indicates that the trans-lactone, the 4β-substituted moiety, and the 4′-demethyl moieties are essential for TOP-II inhibitory activity [9,10]. In recent years, our group and others have found that several analogs with *N*-substitutions at the C4 position show an improved antitumor activity compared with VP-16 [11,12,13,14,15,16,17,18,19,20,21,22,23]. In this study, furfuran amines of 4β*-N*-substituted podophyllotoxin derivatives were designed and synthesized. The antiproliferative activities of the synthesized compounds against human cervical cancer cell line (HeLa), chronic myeloid leukemia cell line (K562) and red leukemia multi-drug resistance cell line (K562/A02) were evaluated and a preliminary SAR study of these compounds is discussed.

## 2. Results and Discussion

### 2.1. Chemistry

**Scheme 1:**
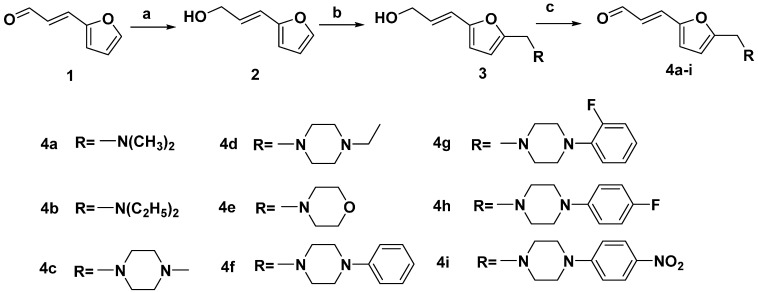
Synthesis of furan intermediates.

The synthesis of compounds **4a**–**i** is outlined in Scheme 1. Treatment of **1** with NaBH_4_ in dry methanol yielded compound **2**. Compound **3** was prepared by means of a Mannich reaction of **2** with a secondary amine in the presence of glacial acetic acid and formaldehyde. Then, compound **3** was reacted with active manganese dioxide to give the intermediates **4a**–**i**.

*Reagents and conditions:* (**a**) NaBH_4_, MeOH, 25 °C, 2 h; (**b**) CH_3_COOH/HCHO/amines, 50 °C, 4 h; (**c**) MnO_2_/CH_2_Cl_2_, 25 °C, 2 h.

**Scheme 2:**
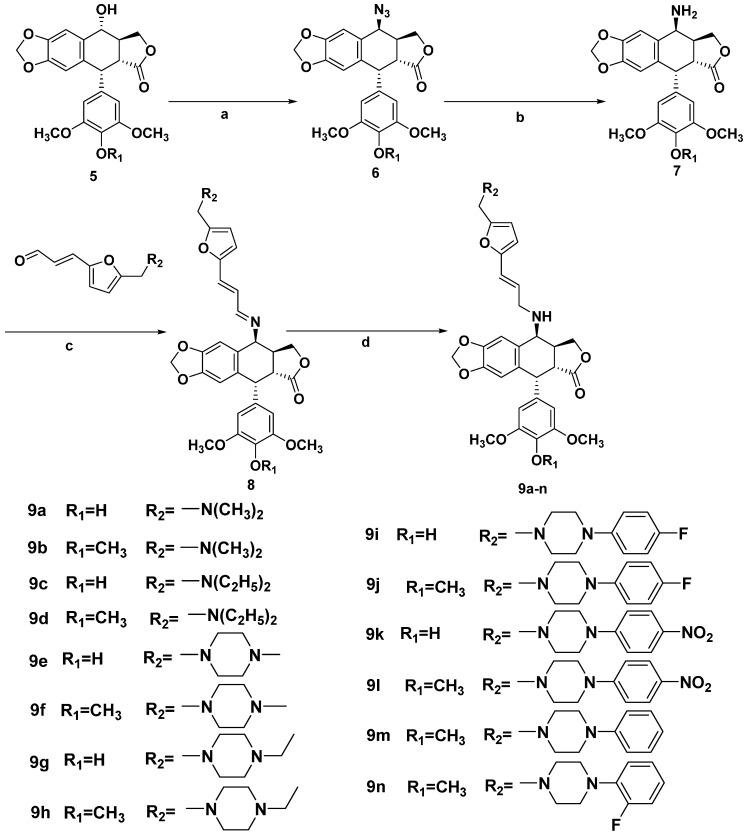
Synthesis of podophyllotoxin derivatives.

The synthetic route (Scheme 2) to the target compounds **9a**–**n** involved the intermediate **7**, which was prepared from **5**. In the presence of sodium azide, compound **6** was derived from **5** [24]. Then, compound **7** was derived from **6** through a reduction of azide. Next, **7** was combined with compounds **4a**–**i**, respectively, in the presence of absolute methanol and a catalytic amount of glacial acetic acid to provide the **8**. Then, reduction of **8** gave compounds **9a**–**n**, respectively. The structures of the intermediates **4a**–**i**, and 14 target compounds were identified by HRMS, ^1^H-NMR, and ^13^C-NMR spectral analysis.

In this paper, the C4-configuration of the novel podophyllotoxin derivatives was deduced from the reaction mechanism as well as evidences from NMR data. The nucleophilic substitution occurring at the C4 position was assumed to follow an SN_1_ mechanism [25]. It was presumed that C4-β-substitution was the main product due to the bulky C1-α-substituted aromatic ring. The configuration of the targeted compounds was identified as C4-β based on their small *J*_3,4_ values, because the *J*_3,4_ value is larger than 10 Hz in C4-α isomer [26].

*Reagents and conditions*: (**a**) NaN_3_, CF_3_COOH, CH_2_Cl_2_, reflux, 4 h; (**b**) Pd/C, HCOONH_4_, EtOAc, reflux, 5 h; (**c**) CH_3_COOH, MeOH, 25 °C, 8 h; (**d**) NaBH_4_, MeOH, 0 °C, 4 h.

### 2.2. Biological Results and Discussion

Cytotoxicities of all target derivatives were evaluated against three human cancer cell lines by the MTT assay. These three cell lines are: HeLa, K562 and K562/A02. The results are summarized in Table 1.

As shown in Table 1, most of compounds exhibited potent antiproliferative activity against all three cell lines with IC_50_ = 1–20 μM. Among them, compounds **9a**, **9e**, **9i** and **9k** were more cytotoxic towards HeLa cells than the positive control, VP-16. Compound **9i** is the strongest antiproliferative activity against HeLa cells (IC_50_ = 0.19 μM). Compound **9e** showed stronger potency against K562 tumor cells than VP-16, whereas Compounds **9a**, **9f**, **9g**, **9i**, **9k**, **9l** and **9m** displayed moderate cytotoxicities in K562 cell lines. Cancer multidrug resistance (MDR) is a common cause of treatment failure in cancer patients. Interestingly, the podophyllotoxin derivatives of **9g**, **9j**, **9k**, **9l** and **9m** showed higher activity toward drug-resistant K562/A02 cells (IC_50_ = 6.89–43.84 μM) than VP-16, indicating a great potential of those derivatives to possess anti-multidrug resistance.

We also deduced the preliminary structure-activity relationships of these compounds. First, the 4′-OH derivatives were more cytotoxic than the corresponding 4′-OMe analogs. This observation is in accord with previously reported activities of closely related structures [11,27]. Second, the introduction of a benzene group (compare **9j** and **9f**, **9l** and **9f**, **9m** and **9f**) such as fluorine substituent at the 4-position of phenyl ring (**9j**), nitro substituent at the 4-position of phenyl ring (**9l**), unsubstituted phenyl ring (**9m**), resulted in a considerably higher increase in cytotoxicity in the MDR cell line, K562/A02, than etoposide. Compound **9l** showed outstanding cytotoxicity towards K562/A02. Our previous results indicate that these derivatives were inhibitors of the expression of MDR-1 in K562/A02 cells [11], having crucial research significance. It was suggested that compound **9l** may overcome MDR by reducing the expression of MDR-1.

**Table 1:** Cytotoxicities of podophyllotoxin derivatives against Hela, K562 and K562/A02.

Compound	IC_50_ (μM) ^a,b^			RF
	HeLa	K562	K562/A02	
**9a**	2.61 ± 0.14	4.19 ± 0.34	118.95 ± 3.21	28.38
**9b**	15.32 ± 0.88	39.31 ± 1.23	159.95 ± 2.14	4.06
**9c**	8.32 ± 0.76	12.46 ± 0.97	91.97 ± 1.23	7.38
**9d**	25.31 ± 1.55	18.72 ± 0.79	>1000	>100
**9e**	3.22 ± 0.78	1.00 ± 0.12	78.66 ± 1.31	78.66
**9f**	22.56 ± 0.88	6.22 ± 0.55	85.75 ± 2.45	13.78
**9g**	25.78 ± 1.02	3.33 ± 0.43	43.94 ± 0.98	13.19
**9h**	>100	>100	>1000	>100
**9i**	0.19 ± 0.01	8.66 ± 0.67	63.79 ± 0.98	7.36
**9j**	10.23 ± 0.75	14.88 ± 0.99	28.29 ± 0.79	1.90
**9k**	0.52 ± 0.01	5.57 ± 0.34	35.32 ± 1.29	6.34
**9l**	7.93 ± 0.59	6.42 ± 0.54	6.89 ± 0.43	1.07
**9m**	7.52 ± 0.67	5.67 ± 0.49	10.31 ± 0.86	1.81
**9n**	NE	NE	NE	NE
**VP-16**	8.27 ± 0.99	4.39 ± 1.21	226.7 ± 4.89	51.64

^a^ The value is the average of three replicates; ^b^ IC_50_: concentration that causes a 50% reduction of cell growth. NE: not evaluated. RF: resistance factor was calculated from the ratio of the growth inhibition constant (IC_50_) of the resistant cell sub-line to that of the parental cell line. VP-16: etoposide—the clinical use of anticancer drugs.

## 3. Experimental Section

### 3.1. Chemistry

Melting points were determined on an electric X-4 digital visual melting point apparatus. The ^1^H-NMR and ^13^C-NMR spectra were obtained using a Bruker ARX instrument (300 MHz, 400 MHz and 600 MHz). Chemical shifts are reported in ppm downfield from internal TMS as standard. HRMS were obtained on Agilent 6210 TOP-MS and are reported as *m*/*z*. Unless otherwise noted, all common reagents and solvents were obtained from commercial suppliers without further purification.

#### 3.1.1. General Procedure for the Synthesis of Compounds **4a**–**i**

Compounds (**4a**–**i**) were synthesized by means of a Mannich reaction. (*E*)-3-(furan-2-yl) acrylaldehyde (1.0 mmol) and NaBH_4_ (2.0 mmol) in dry MeOH (15 mL) were added to a 50 mL dried round-bottom flask. The mixture was reacted at room temperature for 2 h. Then the solvent was evaporated to give the intermediate **2**. A mixture of **2** (1.0 mmol) and the corresponding secondary amine (1.5 mmol) in glacial acetic acid (20 mL) containing formaldehyde (1.5 mmol) was stirred at 50 °C for 4 h. After completion of the reaction was monitored by thin layer chromatography (TLC), the solvent was removed and the residue was added water (15 mL) before neutralization with saturated aqueous NaOH and extraction with ethyl acetate (3 × 30 mL). The combined organic layer was washed with water followed by brine, dried over Na_2_SO_4_, filtered, and concentrated to give compound **3**. To a stirred solution of compound **3** (1.0 mmol) in dry CH_2_Cl_2_ (20 mL) was added active manganese dioxide (10.0 mmol) at room temperature, and the reaction mixture was stirred for 2 h, After the reaction was completed, the mixture was filtered and concentrated to provide a yellow oil and purified by column chromatography on silica gel using petroleum ether-ethyl acetate to afford the yellow solids **4a**–**i** [11].

*(E)-3-(5-((Dimethylamino)methyl)furan-2-yl)acrylaldehyde* (**4a**). Yield: 82%; Colorless oil; ^1^H-NMR (400 MHz, CDCl_3_) δ 9.55 (dd, *J* = 7.9, 1.9 Hz, 1H), 7.13 (dd, *J* = 15.7, 1.8 Hz, 1H), 6.68 (t, *J* = 2.5 Hz, 1H), 6.52 (ddd, *J* = 15.7, 7.9, 1.8 Hz, 1H), 6.32 (t, *J* = 2.5 Hz, 1H), 3.48 (s, 2H), 2.25 (s, 6H). ^13^C-NMR (100 MHz, CDCl_3_) δ 192.7, 156.9, 150.2, 137.7, 125.5, 117.6, 111.7, 55.9, 45.15. HR-ESI-MS *m*/*z*: 180.1141 for [M + H]^+^ (calcd. 180.1025 for C_10_H_14_NO_2_).

*(E)-3-(5-((Diethylamino)methyl)furan-2-yl)acrylaldehyde* (**4b**). Yield: 80%; Colorless oil; ^1^H-NMR (400 MHz, CDCl_3_) δ 9.49 (d, *J* = 8.0 Hz, 1H), 7.08 (d, *J* = 15.6 Hz, 1H), 6.63 (d, *J* = 3.3 Hz, 1H), 6.43 (dd, *J* = 15.6, 8.0 Hz, 1H), 6.25 (d, *J* = 3.3 Hz, 1H), 3.61 (s, 2H), 2.45 (q, *J* = 7.2 Hz, 4H), 0.99 (t, *J* = 7.2 Hz, 6H). ^13^C-NMR (100 MHz, CDCl_3_) δ 192.6, 157.5, 149.8, 137.7, 125.1, 117.7, 111.5, 48.8, 47.1, 12.0. HR-ESI-MS *m*/*z*: 208.1340 for [M + H]^+^ (calcd. 208.1338 for C_12_H_18_NO_2_).

*(E)-3-(5-((4-Methylpiperazin-1-yl)methyl)furan-2-yl)acrylaldehyde* (**4c**). Yield: 78%; Colorless oil; ^1^H-NMR (400 MHz, CDCl_3_) δ 9.51 (d, *J* = 7.9 Hz, 1H), 7.09 (d, *J* = 15.7 Hz, 1H), 6.64 (d, *J* = 3.4 Hz, 1H), 6.47 (dd, *J* = 15.6, 7.9 Hz, 1H), 6.29 (d, *J* = 3.4 Hz, 1H), 3.53 (s, 2H), 2.48 (s, 4H), 2.39 (s, 4H), 2.20 (s, 3H). ^13^C-NMR (100 MHz, CDCl_3_) δ 192.7, 156.1, 150.1, 137.7, 125.5, 117.6, 112.0, 54.9, 54.7, 52.6, 45.9. HR-ESI-MS *m*/*z*: 235.1448 for [M + H]^+^ (calcd. 235.1447 for C_13_H_19_N_2_O_2_).

*(E)-3-(5-((4-Ethylpiperazin-1-yl)methyl)furan-2-yl)acrylaldehyde* (**4d**). Yield: 79%; Colorless oil; ^1^H-NMR (400 MHz, CDCl_3_) δ 9.42 (d, *J* = 7.9 Hz, 1H), 7.02 (d, *J* = 15.6 Hz, 1H), 6.57 (d, *J* = 3.4 Hz, 1H), 6.38 (dd, *J* = 15.6, 7.9 Hz, 1H), 6.21 (d, *J* = 3.4 Hz, 1H), 3.45 (s, 2H), 2.51–2.38 (m, 4H), 2.34 (s, 4H), 2.28–2.21 (m, 2H), 0.90 (t, *J* = 7.2 Hz, 3H). ^13^C-NMR (100 MHz, CDCl_3_) δ 192.4, 155.9, 149.9, 137.5, 125.2, 117.4, 111.8, 54.5, 52.4, 52.4, 51.9, 11.7. HR-ESI-MS *m*/*z*: 249.1607 for [M + H]^+^ (calcd. 249.1603 for C_14_H_21_N_2_O_2_).

*(E)-3-(5-(Morpholinomethyl)furan-2-yl)acrylaldehyde* (**4e**). Yield: 76%; Colorless oil; ^1^H-NMR (400 MHz, CDCl_3_) δ 9.47 (d, *J* = 7.9 Hz, 1H), 7.08 (d, *J* = 15.6 Hz, 1H), 6.63 (d, *J* = 3.4 Hz, 1H), 6.43 (dd, *J* = 15.6, 7.9 Hz, 1H), 6.27 (d, *J* = 3.4 Hz, 1H), 3.62–3.57 (m, 4H), 3.47 (s, 2H), 2.40 (t, *J* = 4.7 Hz, 4H). ^13^C-NMR (100 MHz, CDCl_3_) δ 192.5, 155.7, 150.1, 137.5, 125.4, 117.5, 111.9, 66.6, 55.0, 53.1. HR-ESI-MS *m/z*: 222.1129 for [M + H]^+^ (calcd. 222.1130 for C_12_H_16_NO_3_).

*(E)-3-(5-((4-Phenylpiperazin-1-yl)methyl)furan-2-yl)acrylaldehyde* (**4f**)*.* Yield: 80%; Colorless oil; ^1^H-NMR (400 MHz, CDCl_3_) δ 9.62 (d, *J* = 7.9 Hz, 1H), 7.28 (dd, *J* = 8.8, 7.3 Hz, 2H), 7.20 (d, *J* = 15.7 Hz, 1H), 6.97–6.92 (m, 2H), 6.88 (tt, *J* = 7.3, 1.1 Hz, 1H), 6.76 (d, *J* = 3.4 Hz, 1H), 6.60 (dd, *J* = 15.7, 7.9 Hz, 1H), 6.43 (d, *J* = 3.4 Hz, 1H), 3.69 (s, 2H), 3.26–3.22 (m, 4H), 2.74–2.69 (m, 4H). ^13^C-NMR (100 MHz, CDCl_3_) δ 192.8, 156.1, 151.2, 150.3, 137.8, 129.1, 125.6, 119.8, 117.7, 116.1, 112.1, 54.9, 52.8, 49.1. HR-ESI-MS *m*/*z*: 297.1604 for [M + H]^+^ (calcd. 297.1603 for C_18_H_21_N_2_O_2_).

*(E)-3-(5-((4-(2-Fluorophenyl)piperazin-1-yl)methyl)furan-2-yl)acrylaldehyde* (**4g)***.* Yield: 72%; Colorless oil; ^1^H-NMR (400 MHz, CDCl_3_) δ 9.60 (d, *J* = 7.9 Hz, 1H), 7.18 (d, *J* = 15.6 Hz, 1H), 7.07–6.98 (m, 2H), 6.97–6.90 (m, 2H), 6.73 (d, *J* = 3.4 Hz, 1H), 6.58 (dd, *J* = 15.6, 7.9 Hz, 1H), 6.40 (d, *J* = 3.4 Hz, 1H), 3.68 (s, 2H), 3.15–3.11(m, 4H), 2.73–2.67 (m, 4H). ^13^C-NMR (100 MHz, CDCl_3_) δ 192.9, 150.4, 137.9, 125.8, 124.6, 122.6, 119.0, 117.7, 116.3, 116.1, 112.3, 77.4, 77.1, 76.8, 55.0, 53.0, 50.5. HR-ESI-MS *m*/*z*: 315.1508 for [M + H]^+^ (calcd. 315.1509 for C_18_H_20_FN_2_O_2_).

*(E)-3-(5-((4-(4-Fluorophenyl)piperazin-1-yl)methyl)furan-2-yl)acrylaldehyde* (**4h**)*.* Yield: 74%; Colorless oil; ^1^H-NMR (400 MHz, CDCl_3_) δ 9.60 (d, *J* = 7.9 Hz, 1H), 7.18 (d, *J* = 15.7 Hz, 1H), 6.97–6.92 (m, 2H), 6.90–6.84 (m, 2H), 6.73 (d, *J* = 3.4 Hz, 1H), 6.57 (dd, *J* = 15.7, 7.9 Hz, 1H), 6.39 (d, *J* = 3.4 Hz, 1H), 3.67 (s, 2H), 3.16–3.11 (m, 4H), 2.71–2.66 (m, 4H). ^13^C-NMR (100 MHz, CDCl_3_) δ 192.9, 156.1, 150.4, 147.9, 137.8, 125.7, 118.0, 117.7, 115.7, 115.5, 112.2, 54.9, 52.9, 50.2. HR-ESI-MS *m*/*z*: 315.1511 for [M + H]^+^ (calcd. 315.1509 for C_18_H_20_FN_2_O_2_).

*(E)-3-(5-((4-(4-Nitrophenyl)piperazin-1-yl)methyl)furan-2-yl)acrylaldehyde* (**4i**). Yield: 75%; Yellow oil; ^1^H-NMR (400 MHz, CDCl_3_) δ 9.50 (d, *J* = 7.9 Hz, 1H), 7.96 (d, *J* = 9.6 Hz, 2H), 7.11 (d, *J* = 15.6 Hz, 1H), 6.70 (d, *J* = 9.6 Hz, 2H), 6.68 (d, *J* = 3.3 Hz, 1H), 6.45 (dd, *J* = 15.6, 7.9 Hz, 1H), 6.33 (d, *J* = 3.3 Hz, 1H), 3.58 (s, 2H), 3.38–3.30 (m, 4H), 2.60–2.55 (m, 4H). ^13^C-NMR (100 MHz, CDCl_3_) δ 192.5, 155.4, 154.5, 150.1, 138.0, 137.6, 125.6, 125.3, 117.6, 112.4, 112.1, 54.4, 52.0, 46.7. HR-ESI-MS *m*/*z*: 342.1457 for [M + H]^+^ (calcd. 342.1454 for C_18_H_20_N_3_O_4_).

#### 3.1.2. General Procedure for the Synthesis of Compounds **9a**–**n**

To a stirred solution of **5** (10 mmol) in dry CH_2_Cl_2_ (50 mL), NaN_3_ (40 mmol) in dry CH_2_Cl_2_ (10 mL) was added carefully. CF_3_COOH (10 mL) was added into the solution dropwise at 0 °C. After stirring for 1 h at room temperature, the mixture was refluxed for 4 h. Saturated aqueous NaHCO_3_ was added to adjust the pH value to 7. The organic phase was separated and dried with anhydrous Na_2_SO_4_ and concentrated. The residue was crystallized from CH_2_Cl_2_/acetic ether (1:1) to give a **6**. To a solution of **6** (10 mmol) in ethyl acetate (50 mL), 10% Pd/C (1.00 g) and HCOONH_4_ (40 mmol) were added. The mixture was refluxed for 5 h and filtered. The filtrate was washed with saturated brine three times and concentrated to give white compound **7** [11].

A mixture of the appropriate intermediate **4a**–**i** (1.5 mmol), **7** (1.0 mmol), and glacial acetic acid (60 μL) was stirred in dry MeOH (15 mL) for 8 h at room temperature. Then NaBH_4_ (4 mmol) was added and the mixture was stirred for 4 h at 0 °C. The reaction mixture was neutralized with 1 M HCl, and extracted with CH_2_Cl_2_ (3 × 30 mL). The combined organic fractions were evaporated. The residue was separated by column chromatography on silica gel with petroleum ether-acetic ether to afford compounds **9a**–**n** (see the supplementary information).

*4β-N-[(E)-(5-((Dimethylamino)methyl)furan-2-yl)prop-2-en-1-amine]-4′-demethyl-4-desoxy-podophyllotoxin* (**9a**). Yield: 77%; white powder solid; mp: 229–230 °C; ${\left[\alpha \right]}_{D}^{25}$ −52° (*c* 0.1, CH_3_CN); ^1^H-NMR (300 MHz, CDCl_3_) δ 6.76 (s, 1H), 6.46 (s, 1H), 6.37 (d, *J* = 15.9 Hz, 1H), 6.27 (s, 2H), 6.26–6.20 (m, 2H), 6.18 (d, *J* = 3.4 Hz, 1H), 5.95 (d, *J* = 1.3 Hz, 1H), 5.93 (d, *J* = 1.3 Hz, 1H), 4.53 (d, *J* = 5.2 Hz, 1H), 4.34–4.23 (m, 2H), 3.91 (d, *J* = 3.9 Hz, 1H), 3.76 (s, 6H), 3.55 (s, 2H), 3.43–3.35 (m, 2H), 3.30 (dd, *J* = 13.8, 5.2 Hz, 1H), 2.83–2.70 (m, 1H), 2.34 (s, 6H). ^13^C-NMR (150 MHz, CDCl_3_) δ 175.4, 147.7, 147.3, 146.3, 133.9, 132.5, 131.7, 131.1, 120.0, 110.2, 108.5, 108.3, 107.9, 101.3, 68.5, 56.5, 56.4, 55.6, 52.1, 43.5, 41.4, 38.6, 29.7. HR-ESI-MS *m*/*z*: 563.2339 for [M + H]^+^ (calcd. 563.2393 for C_31_H_34_N_2_O_8_).

*4β-N-[(E)-(5-((Dimethylamino)methyl)furan-2-yl)prop-2-en-1-amine]-4-desoxy-podophyllotoxin* (**9b**)*.* Yield: 72%; white powder solid; mp: 230–231 °C; ${\left[\alpha \right]}_{D}^{25}$ −53° (*c* 0.1, CH_3_CN); ^1^H-NMR (300 MHz, CDCl_3_) δ 6.77 (s, 1H), 6.46 (s, 1H), 6.36 (d, *J* = 15.9 Hz, 1H), 6.26 (s, 2H), 6.23–6.14 (m, 3H), 5.95 (d, *J* = 1.4 Hz, 1H), 5.93 (d, *J* = 1.4 Hz, 1H), 4.53 (d, *J* = 5.2 Hz, 1H), 4.31 (dd, *J* = 9.1, 3.3 Hz, 2H), 3.92 (d, *J* = 3.9 Hz, 1H), 3.79 (s, 3H), 3.73 (s, 6H), 3.50 (s, 2H), 3.43–3.34 (m, 2H), 3.34–3.27 (m, 1H), 2.81–2.72 (m, 1H), 2.30 (s, 6H). ^13^C-NMR (150 MHz, CDCl_3_) δ 175.4, 152.4, 147.7, 147.3, 137.1, 135.6, 132.5, 131.5, 126.5, 120.2, 110.2, 108.4, 108.4, 108.3, 101.4, 68.6, 60.7, 56.2, 55.6, 52.2, 44.3, 43.7, 41.3, 38.7. HR-ESI-MS *m*/*z*: 577.2515 for [M + H]^+^ (calcd. 577.2550 for C_32_H_36_N_2_O_8_).

*4β-N-[(E)-(5-((Diethylamino)methyl)furan-2-yl)prop-2-en-1-amine]-4′-demethyl-4-desoxy-podophyllotoxin* (**9c**). Yield: 70%; white powder solid; mp: 233–235 °C; ${\left[\alpha \right]}_{D}^{25}$ −50° (*c* 0.1, CH_3_CN); ^1^H-NMR (600 MHz, CDCl_3_) δ 6.80 (s, 1H), 6.50 (s, 1H), 6.39 (d, *J* = 15.8 Hz, 1H), 6.31 (s, 2H), 6.20 (s, 2H), 6.20–6.16 (m, 1H,), 5.98 (s, 1H,), 5.96 (s, 1H), 4.55 (d, *J* = 5.2 Hz, 1H), 4.35–4.29 (m, 2H), 3.94 (d, *J* = 3.9 Hz, 1H), 3.79 (s, 6H), 3.73 (s, 2H), 3.52 (dd, *J* = 14.4, 7.0 Hz, 1H), 3.43 (dd, *J* = 14.4, 7.0 Hz, 1H), 3.33 (dd, *J* = 13.8, 5.2 Hz, 1H), 2.84–2.76 (m, 1H), 2.59 (q, *J* = 7.1 Hz, 4H), 1.14 (t, *J* = 7.1 Hz, 6H). ^13^C-NMR (150 MHz, CDCl_3_) δ 175.5, 151.7, 147.7, 147.3, 146.3, 133.9, 132.6, 131.7, 131.1, 125.7, 120.6, 110.2, 108.4, 108.4, 107.9, 101.3, 68.6, 56.4, 55.5, 52.2, 48.5, 46.9, 43.5, 41.4, 38.6, 11.9. HR-ESI-MS *m*/*z:* 591.2719 for [M + H]^+^ (calcd. 591.2706 for C_33_H_38_N_2_O_8_).

*4β-N-[(E)-(5-((Diethylamino)methyl)furan-2-yl)prop-2-en-1-amine]-4-desoxy-podophyllotoxin* (**9d**)*.* Yield: 68%; white powder solid; mp: 236–237 °C; ${\left[\alpha \right]}_{D}^{25}$ −59° (*c* 0.1, CH_3_CN); ^1^H-NMR (300 MHz, CDCl_3_) δ 6.76 (s, 1H), 6.46 (s, 1H), 6.39–6.32 (m, 1H), 6.26 (s, 2H), 6.16 (s, 2H), 6.16–6.09 (m, 1H), 5.95 (d, *J* = 1.4 Hz, 1H), 5.93 (d, *J* = 1.4 Hz, 1H), 4.53 (d, *J* = 5.2 Hz, 1H), 4.31 (dd, *J* = 9.0, 2.0 Hz, 2H), 3.92 (d, *J* = 3.9 Hz, 1H), 3.79 (s, 3H), 3.73 (s, 6H), 3.69 (s, 2H), 3.55–3.45 (m, 1H), 3.44–3.37 (m, 1H), 3.36–3.26 (m, 1H), 2.82–2.72 (m, 1H), 2.57 (q, *J* = 7.1 Hz, 4H), 1.11 (t, *J* = 7.1 Hz, 6H). ^13^C-NMR (150 MHz, CDCl_3_) δ 175.4, 152.4, 147.6, 147.3, 137.0, 135.6, 132.5, 131.5, 125.9, 120.4, 110.1, 108.4, 108.4, 108.2, 101.3, 77.2, 77.0, 76.8, 68.5, 60.7, 56.2, 55.5, 52.2, 46.8, 43.7, 41.3, 38.6, 11.6. HR-ESI-MS *m*/*z*: 605.2869 for [M + H]^+^ (calcd. 605.2863 for C_34_H_40_N_2_O_8_).

*4β-N-[(E)-(5-((4-Methylpiperazin-1-yl)methyl)furan-2-yl)prop-2-en-1-amine]-4′-demethyl-4-desoxy-podophyllotoxin* (**9e**). Yield: 71%; white powder solid; mp: 238–239 °C; ${\left[\alpha \right]}_{D}^{25}$ −61° (*c* 0.1, CH_3_CN); ^1^H-NMR (300 MHz, CDCl_3_) δ 6.76 (s, 1H), 6.46 (s, 1H), 6.40–6.31 (m, 2H), 6.27 (s, 2H), 6.24–6.13 (m, 2H), 5.94 (dd, *J* = 6.9, 1.3 Hz, 2H), 4.52 (d, *J* = 5.2 Hz, 1H), 4.34–4.26 (m, 2H), 3.91 (d, *J* = 4.0 Hz, 1H), 3.76 (s, 6H), 3.58 (s, 2H), 3.51 (dd, *J* = 14.7, 7.2 Hz, 1H), 3.38 (dd, *J* = 14.7, 7.2 Hz, 1H), 3.30 (dd, *J* = 13.8, 5.1 Hz, 1H), 2.85–2.77 (m, 1H), 2.78–2.52 (m, 8H), 2.42 (s, 3H). ^13^C-NMR (150 MHz, CDCl_3_) δ 175.5, 152.0, 151.1, 147.7, 147.2, 146.3, 133.9, 132.6, 131.7, 131.1, 126.0, 120.5, 110.7, 110.2, 108.4, 108.4, 107.9, 101.3, 68.6, 56.4, 55.5, 54.8, 54.7, 52.2, 45.7, 43.5, 41.4, 38.6, 29.7. HR-ESI-MS *m*/*z*: 618.2823 for [M + H]^+^ (calcd. 618.2815 for C_34_H_39_N_3_O_8_).

*4β-N-[(E)-(5-((4-methylpiperazin-1-yl)methyl)furan-2-yl)prop-2-en-1-amine]-4-desoxy-podophyllotoxin* (**9f**). Yield: 74%; white powder solid; mp: 239–241 °C; ${\left[\alpha \right]}_{D}^{25}$ −63° (*c* 0.1, CH_3_CN); ^1^H-NMR (300 MHz, CDCl_3_) δ 6.76 (s, 1H), 6.47 (s, 1H), 6.35 (d, *J* = 15.9 Hz, 1H), 6.26 (s, 2H), 6.22–6.11 (m, 3H), 5.96–5.94 (m, 1H), 5.94–5.92 (m, 1H), 4.53 (d, *J* = 5.2 Hz, 1H), 4.36–4.25 (m, 2H), 3.91 (d, *J* = 3.9 Hz, 1H), 3.79 (s, 3H), 3.73 (s, 6H), 3.57 (s, 2H), 3.50 (dd, *J* = 14.6, 7.0 Hz, 1H), 3.44–3.35 (m, 1H), 3.32 (dd, *J* = 13.8, 5.3 Hz, 1H), 2.86–2.73 (m, 1H), 2.68–2.42 (m, 8H), 2.31 (s, 3H). ^13^C-NMR (150 MHz, CDCl_3_) δ 175.4, 152.4, 152.0, 147.7, 147.3, 137.0, 135.6, 132.6, 131.5, 126.0, 120.5, 110.8, 110.2,108.4, 108.4, 108.2, 101.3, 68.6, 60.7, 56.2, 55.5, 54.7, 54.6, 52.2, 43.7, 41.3, 38.7, 31.9. HR-ESI-MS *m*/*z*: 632.2982 for [M + H]^+^ (calcd. 632.2972 for C_35_H_41_N_3_O_8_).

*4β-N-[(E)-(5-((4-Ethylpiperazin-1-yl)methyl)furan-2-yl)prop-2-en-1-amine]-4′-demethyl-4-desoxy-podophyllotoxin* (**9g**). Yield: 78%; white powder solid; mp: 241–243 °C; ${\left[\alpha \right]}_{D}^{25}$ −64° (*c* 0.1, CH_3_CN); ^1^H-NMR (300 MHz, CDCl_3_) δ 6.76 (s, 1H), 6.46 (s, 1H), 6.34 (d, *J* = 15.7 Hz, 1H), 6.26 (s, 2H), 6.22–6.09 (m, 3H), 5.93 (dd, *J* = 5.7, 1.4 Hz, 2H), 4.51 (d, *J* = 5.2 Hz, 1H), 4.32–4.23 (m, 2H), 3.91 (d, *J* = 3.9 Hz, 1H), 3.73 (s, 6H), 3.56 (s, 2H), 3.47 (dd, *J* = 14.7, 5.7 Hz, 1H), 3.37 (dd, *J* = 14.6, 5.7 Hz, 1H), 3.29 (dd, *J* = 13.8, 5.2 Hz, 1H), 2.85–2.71 (m, 1H), 2.70–2.45 (m, 8H), 2.49–2.40 (m, 2H), 1.08 (t, *J* = 7.2 Hz, 3H). ^13^C-NMR (150 MHz, CDCl_3_) δ 175.5, 152.0, 147.7, 147.2, 146.3, 133.9, 132.6, 131.7, 131.1, 126.0, 120.5, 110.8, 110.2, 108.4, 108.4, 107.9, 101.3, 68.6, 56.4, 55.5, 54.6, 52.4, 52.2, 52.2, 43.5, 41.4, 38.6. 11.5. HR-ESI-MS *m*/*z*: 632.2979 for [M + H]^+^ (calcd. 632.2972 for C_35_H_41_N_3_O_8_).

*4β-N-[(E)-(5-((4-Ethylpiperazin-1-yl)methyl)furan-2-yl)prop-2-en-1-amine]-4-desoxy-podophyllotoxin* (**9h**). Yield: 76%; white powder solid; mp: 243–244 °C; ${\left[\alpha \right]}_{D}^{25}$ −65° (*c* 0.1, CH_3_CN); ^1^H-NMR (300 MHz, CDCl_3_) δ 6.77 (s, 1H), 6.46 (s, 1H), 6.35 (d, *J* = 15.8 Hz, 1H), 6.26 (s, 2H), 6.21–6.12 (m, 3H), 5.95 (d, *J* = 1.4 Hz, 1H), 5.93 (d, *J* = 1.4 Hz, 1H), 4.53 (d, *J* = 5.2 Hz, 1H), 4.35–4.27 (m, 2H), 3.91 (d, *J* = 3.9 Hz, 1H), 3.79 (s, 3H), 3.73 (s, 6H), 3.57 (s, 2H), 3.50 (dd, *J* = 14.4, 7.1 Hz, 1H), 3.44–3.36 (m, 1H,), 3.35–3.27 (m, 1H), 2.82–2.74 (m, 1H), 2.70–2.45 (m, 8H), 2.43 (q, *J* = 7.2 Hz, 2H,), 1.08 (t, *J* = 7.2 Hz, 3H). ^13^C-NMR (150 MHz, CDCl_3_) δ 175.4, 152.4, 151.9, 151.2, 147.6, 147.3, 137.0, 135.6, 132.6, 131.5, 125.9, 120.5, 110.7, 110.2, 108.4, 108.4, 108.2, 101.3, 77.2, 76.9, 76.7, 68.6, 60.7, 56.2, 55.5, 54.6, 52.5, 52.2, 52.1, 43.7, 41.3, 38.6, 11.6. HR-ESI-MS *m*/*z*: 646.3172 for [M + H]^+^ (calcd. 646.3128 for C_36_H_43_N_3_O_8_).

*4β-N-[(E)-(5-((4-(4-Fluorophenyl)piperazin-1-yl)methyl)furan-2-yl)prop-2-en-1-amine]-4′-demethyl-4-desoxy-podophyllotoxin* (**9i**). Yield: 74%; white powder solid; mp: 250–251 °C; ${\left[\alpha \right]}_{D}^{25}$ −21° (*c* 0.1, CH_3_CN); ^1^H-NMR (600 MHz, CDCl_3_) δ 6.96–6.92 (m, 2H), 6.88–6.84 (m, 2H), 6.77 (s, 1H), 6.47 (s, 1H), 6.38 (d, *J* = 15.8 Hz, 1H), 6.28 (s, 2H), 6.27–6.24 (m, 1H), 6.23–6.18 (m, 2H), 5.93 (d, *J* = 1.4 Hz, 1H), 5.91 (d, *J* = 1.4 Hz, 1H), 4.53 (d, *J* = 5.2 Hz, 1H), 4.34–4.26 (m, 2H), 3.91 (d, *J* = 3.9 Hz, 1H), 3.76 (s, 6H), 3.68–3.60 (m, 2H), 3.51 (dd, J = 14.5, 7.1 Hz, 1H), 3.43–3.37 (m, 1H), 3.30 (dd, *J* = 13.8, 5.2 Hz, 1H), 3.21–3.09 (m, 4H), 2.81–2.75 (m, 1H), 2.73–2.64 (m, 4H). ^13^C-NMR (150 MHz, CDCl_3_) δ 175.4, 147.6, 147.2, 146.3, 133.9, 132.5, 131.7, 131.1, 120.4, 117.9, 115.5, 115.4, 110.2, 108.4, 108.3, 107.9, 101.3, 77.1, 76.9, 76.7, 68.5, 56.4, 55.5, 52.5, 52.2, 50.0, 43.5, 41.4, 38.6. HR-ESI-MS *m*/*z*: 698.2896 for [M + H]^+^ (calcd. 698.2878 for C_39_H_40_FN_3_O_8_).

*4β-N-[(E)-(5-((4-(4-Fluorophenyl)piperazin-1-yl)methyl)furan-2-yl)prop-2-en-1-amine]-4-desoxy-podophyllotoxin* (**9j**). Yield: 73%; white powder solid; mp: 252–253 °C; ${\left[\alpha \right]}_{D}^{25}$ −31° (*c* 0.1, CH_3_CN); ^1^H-NMR (600 MHz, CDCl_3_) δ 6.97–6.92 (m, 2H), 6.88–6.83 (m, 2H), 6.77 (s, 1H), 6.47 (s, 1H), 6.38 (d, *J* = 15.9 Hz, 1H), 6.27 (s, 2H), 6.26–6.22 (m, 1H), 6.22–6.17 (m, 1H), 6.20 (d, *J* = 3.2 Hz, 1H), 5.92 (d, *J* = 1.4 Hz, 1H), 5.91 (d, *J* = 1.4 Hz, 1H), 4.53 (d, *J* = 5.2 Hz, 1H), 4.34–4.27 (m, 2H), 3.92 (d, *J* = 3.9 Hz, 1H), 3.79 (s, 3H), 3.73 (s, 6H), 3.66 (s, 2H), 3.53–3.48 (m, 1H), 3.39 (dd, *J* = 14.4, 5.7 Hz, 1H), 3.32 (dd, *J* = 13.8, 5.2 Hz, 1H), 3.16 (t, *J* = 4.9 Hz, 4H), 2.82–2.76 (m, 1H), 2.74–2.68 (m, 4H). ^13^C-NMR (150 MHz, CDCl_3_) δ 175.3, 152.4, 147.7, 147.3, 135.6, 132.5, 131.5, 120.4, 117.9, 117.8, 115.5, 115.4, 111.1, 110.2, 108.4, 108.4, 108.2, 101.3, 68.5, 60.7, 56.2, 55.5, 52.5, 52.2, 49.9, 43.7, 41.3, 38.6. HR-ESI-MS *m*/*z*: 712.3019 for [M + H]^+^ (calcd. 712.3034 for C_40_H_42_FN_3_O_8_).

*4β-N-[(E)-(5-((4-(4-Nitrophenyl)piperazin-1-yl)methyl)furan-2-yl)prop-2-en-1-amine]-4′-demethyl-4-desoxy-podophyllotoxin* (**9k**). Yield: 64%; yellow powder solid; mp: 254–255 °C; ${\left[\alpha \right]}_{D}^{25}$ −32° (*c* 0.1, CH_3_CN); ^1^H-NMR (600 MHz, CDCl_3_) δ 8.09 (d, *J* = 9.4 Hz, 2H), 6.79 (d, *J* = 9.4 Hz, 2H), 6.77 (s, 1H), 6.47 (s, 1H), 6.38 (d, *J* = 15.9 Hz, 1H), 6.27 (s, 2H), 6.22 (d, *J* = 3.2 Hz, 1H), 6.21–6.16 (m, 2H), 5.94 (d, *J* = 1.4 Hz, 1H), 5.92 (d, *J* = 1.4 Hz, 1H), 4.52 (d, *J* = 5.2 Hz, 1H), 4.31–4.26 (m, 2H), 3.91 (d, *J* = 3.9 Hz, 1H), 3.76 (s, 6H,), 3.62 (s, 2H), 3.53 (dd, *J* = 14.4, 5.6 Hz, 1H), 3.44 (t, *J* = 5.1 Hz, 4H), 3.38 (dd, *J* = 14.4, 5.6 Hz, 1H), 3.30 (dd, *J* = 13.8, 5.2 Hz, 1H), 2.81–2.74 (m, 1H), 2.65 (t, *J* = 5.1 Hz, 4H). ^13^C-NMR (150 MHz, CDCl_3_) δ 175.4, 154.7, 152.1, 150.7, 147.6, 147.2, 146.3, 138.4, 133.9, 132.5, 131.7, 131.1, 126.2, 125.9, 120.3, 112.6, 110.9, 110.2, 108.4, 108.3, 107.9, 101.3, 68.5, 56.4, 55.6, 54.7, 52.2, 52.0, 46.9, 43.5, 41.4, 38.6. HR-ESI-MS *m*/*z*: 725.2831 for [M + H]^+^ (calcd. 725.2823 for C_39_H_40_N_4_O_10_).

*4β-N-[(E)-(5-((4-(4-Nitrophenyl)piperazin-1-yl)methyl)furan-2-yl)prop-2-en-1-amine]-4-desoxy-podophyllotoxin* (**9l**). Yield: 62%; yellow powder solid; mp: 256–257 °C; ${\left[\alpha \right]}_{D}^{25}$ −35° (*c* 0.1, CH_3_CN); ^1^H-NMR (600 MHz, CDCl_3_) δ 8.08 (d, *J* = 9.4 Hz, 2H), 6.78 (d, *J* = 9.4 Hz, 2H), 6.77 (s, 1H), 6.46 (s, 1H), 6.37 (d, *J* = 16.0 Hz, 1H), 6.26 (s, 2H), 6.21 (d, *J* = 3.3 Hz, 1H), 6.20–6.16 (m, 2H), 5.92 (d, *J* = 1.4 Hz, 1H), 5.91 (d, *J* = 1.4 Hz, 1H), 4.52 (d, *J* = 5.2 Hz, 1H), 4.33–4.26 (m, 2H), 3.91 (d, *J* = 3.9 Hz, 1H), 3.77 (s, 3H), 3.72 (s, 6H), 3.61 (s, 2H), 3.55–3.49 (m, 1H), 3.45–3.41 (m, 4H), 3.40–3.35 (m, 1H), 3.31 (dd, *J* = 13.8, 5.3 Hz, 1H), 2.82–2.74 (m, 1H), 2.64 (t, *J* = 5.2 Hz, 4H). ^13^C-NMR (150 MHz, CDCl_3_) δ 175.4, 154.7, 152.4, 152.1, 150.7, 147.6, 147.3, 138.3, 135.6 132.5, 131.5, 126.2, 125.8, 120.3, 112.5, 110.9, 110.2, 108.4, 108.2, 101.3, 77.2, 77.0, 76.8, 68.6, 60.7, 56.2, 55.6, 54.6, 52.2, 52.0, 46.9, 43.7, 41.3, 38.6. HR-ESI-MS *m*/*z*: 739.2992 for [M + H]^+^ (calcd. 739.2979 for C_40_H_42_N_4_O_10_).

*4β-N-[(E)-(5-((4-Phenylpiperazin-1-yl)methyl)furan-2-yl)prop-2-en-1-amine]-4-desoxy-podophyllotoxin* (**9m**). Yield: 71%; white powder solid; mp: 248–249 °C; ${\left[\alpha \right]}_{D}^{25}$ −33° (*c* 0.1, CH_3_CN); ^1^H-NMR (600 MHz, CDCl_3_) δ 7.28–7.21 (m, 2H), 6.91 (d, *J* = 7.5 Hz, 2H), 6.85 (t, *J* = 7.5 Hz, 1H), 6.44 (s, 1H), 6.37 (s, 1H), 6.25 (s, 2H), 6.24 (s, 1H), 6.24–6.18 (m, 2H), 6.21 (d, *J* = 3.0 Hz, 1H), 5.87 (s, 2H), 4.52 (d, *J* = 5.2 Hz, 1H), 4.32–4.23 (m, 2H), 3.92 (d, *J* = 4.0 Hz, 1H), 3.90 (d, *J* = 15.1 Hz, 1H), 3.79 (s, 3H), 3.72 (s, 6H), 3.67–3.62 (m, 2H), 3.62 (d, *J* = 15.1 Hz, 1H), 3.32 (dd, *J* = 13.8, 5.3 Hz, 1H), 3.27–3.20 (m, 4H), 2.80–2.73 (m, 1H), 2.72–2.64 (m, 4H). ^13^C-NMR (150 MHz, CDCl_3_) δ 175.4, 154.8, 152.4, 147.7, 147.3, 137.0, 135.6, 132.5, 131.5, 124.4, 118.9, 116.1, 116.0, 110.2, 108.4, 108.2, 101.3, 77.1, 76.9, 76.7, 68.5, 60.7, 56.2, 55.4, 52.5, 52.2, 43.7, 41.3, 38.7. HR-ESI-MS *m*/*z*: 694.3128 for [M + H]^+^ (calcd. 694.3128 for C40H44N3O8).

*4β-N-[(E)-(5-((4-(2-Fluorophenyl)piperazin-1-yl)methyl)furan-2-yl)prop-2-en-1-amine]-4-desoxy-podophyllotoxin* (**9n**). Yield: 69%; white powder solid; mp: 250–251 °C; ${\left[\alpha \right]}_{D}^{25}$ −34° (*c* 0.1, CH_3_CN); ^1^H-NMR (600 MHz, CDCl_3_) δ 7.07–6.98 (m, 2H), 6.97–6.90 (m, 2H), 6.77 (s, 1H), 6.47 (s, 1H), 6.38 (d, *J* = 15.9 Hz, 1H), 6.27 (s, 2H), 6.24 (s, 1H), 6.24–6.21 (m, 1H), 6.20 (d, *J* = 3.6 Hz, 1H), 5.92 (s, 1H), 5.92 (s, 1H), 4.54 (d, *J* = 5.3 Hz, 1H), 4.35–4.28 (m, 2H), 3.92 (d, *J* = 3.9 Hz, 1H), 3.79 (s, 3H), 3.73 (s, 6H), 3.71–3.65 (m, 2H) 3.52–3.46 (m, 1H), 3.44–3.38 (m, 1H), 3.32 (dd, *J* = 13.8, 5.3 Hz, 1H), 3.20–3.09 (m, 4H), 2.82–2.76 (m, 1H), 2.78–2.68 (m, 4H). ^13^C-NMR (150 MHz, CDCl_3_) δ 175.4, 154.8, 152.4, 147.7, 147.3, 137.0, 135.6, 132.5, 131.5, 124.4, 118.9, 116.1, 116.0, 110.2, 108.4, 108.2, 101.3, 77.1, 76.9, 76.7, 68.5, 60.7, 56.2, 55.4, 52.5, 52.2, 43.7, 41.3, 38.7. HR-ESI-MS *m*/*z*: 712.3027 for [M + H]^+^ (calcd. 712.3034 for C_40_H_42_FN_3_O_8_).

### 3.2. Evaluation of the Biological Activity

The antiproliferative activity of compounds **9a**–**n** was evaluated with human cervical cancer cell line (HeLa), chronic myeloid leukemia cell line (K562) and leukemia multi-drug resistance cell line (K562/A02) by the MTT method *in vitro*, with etoposide (VP-16) as positive control. The three tumor cell lines were cultured in RPMI-1640 containing 10% FBS, 2 mmol∙L^−1^ glutamine, 100 U∙mL^−1^ penicillin, and 100 µg∙mL^−1^ streptomycin at 37 °C in a humidified atmosphere with 5% CO_2_. The cells were seeded at a density of 5 × 103 cells/well in 96-well plates and allowed to attach for 24 h. The thiazolyl blue tetrazolium bromide (MTT) assay was performed to quantify cell viability following treatment with the synthetic compounds or reference compound etoposide (VP-16) [28]. After 48 h, 20 μL MTT (5 mg∙mL^−1^) solution was added for 4 h at 37 °C. Then, the supernatant was discarded and dimethylsulfoxide (150 μL) was added to dissolve the formazan product. The intensity was measured at a wavelength of 490 nm.

## 4. Conclusions

In summary, a new series of podophyllotoxin derivatives were prepared. Most of compounds showed potent antiproliferative activity against all three cancer cell lines. Compounds **9g**, **9j**, **9k**, **9l** and **9m** exhibited more potent activity against the MDR cell line (K562/A02) as compared with VP-16. Among them, 4β*-N*-[(*E*)-(5-((4-(4-nitrophenyl)piperazin-1-yl)methyl)furan-2-yl)prop2-en-1-amine]-4-desoxy-podophyllotoxin (**9l**), was the most promising compound against the tested cell lines. The initial SARs showed that variations in the substituents on the phenyl ring had a significant impact on the cytotoxicity.

## Supplementary Materials

Supplementary materials can be accessed at: http://www.mdpi.com/1420-3049/20/07/12266/s1.
