# Supplementary material for: Synthesis and Evaluation of New Podophyllotoxin Derivatives with in Vitro Anticancer Activity
Source: Molecules. 2015 Jul 6;20(7):12266–79. doi: 10.3390/molecules200712266 (PMC6332074; doi:10.3390/molecules200712266)
Supplement: Supplementary file 1 [file molecules-20-12266-s001.pdf]

Supplementary Material

Copies of ^{1}H-, ^{13}C-NMR and Mass (HRMS) spectra for all new compounds.

4β-N-[(E)-(5-((Dimethylamino)methyl)furan-2-yl)prop-2-en-1-amine]-4'-demethyl-4-desoxy-podophyllotoxin (**9a**)

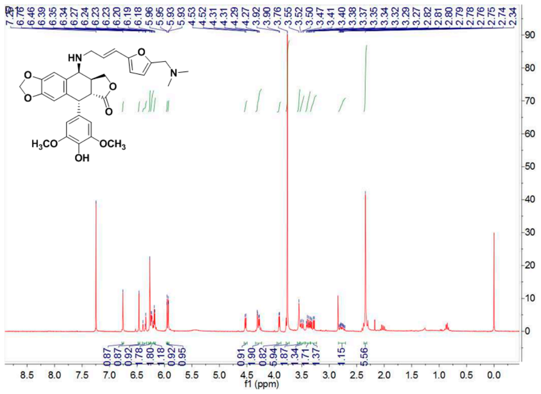

Figure S1. ^{1}H-NMR of compound **9a**.

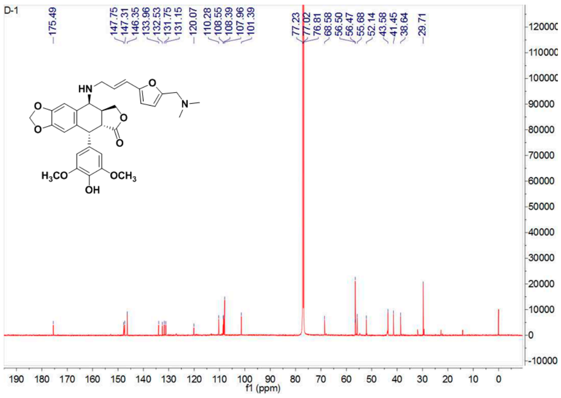

Figure S2. ^{13}C-NMR of compound **9a**.

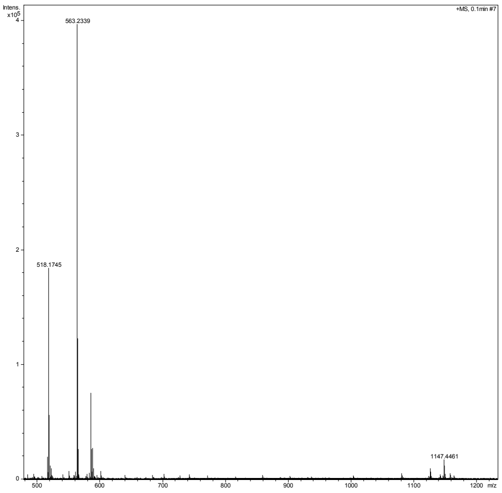

Figure S3. HR-ESI-MS spectra of compound **9a**.

4β-N-[(E)-(5-((Dimethylamino)methyl)furan-2-yl)prop-2-en-1-amine]-4-desoxy-podophyllotoxin (**9b**)

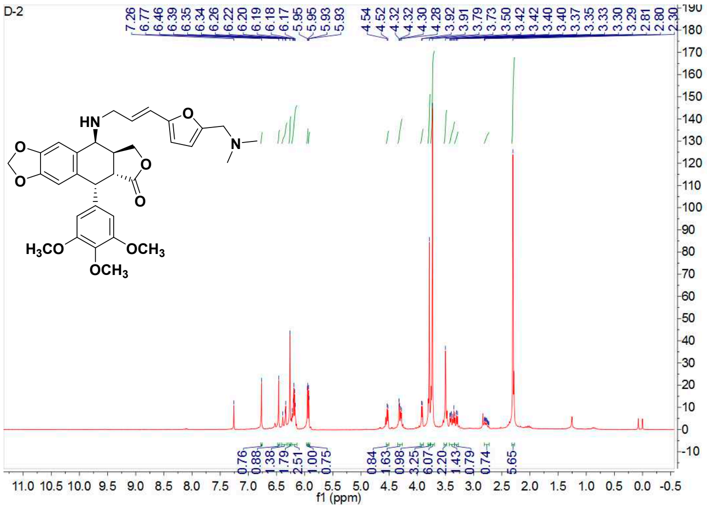

Figure S4. ^{1}H-NMR of compound **9b**.

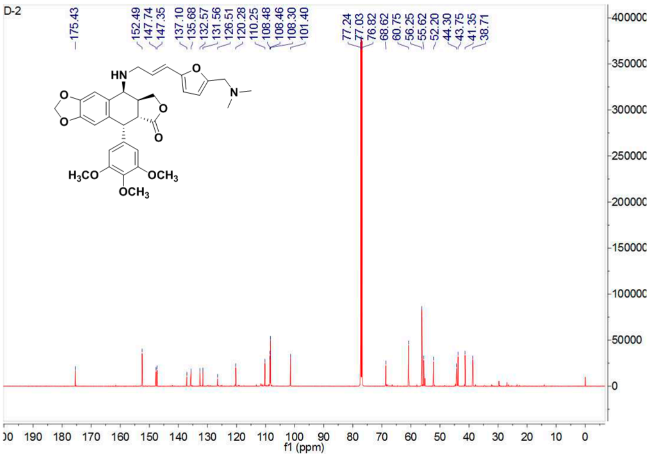

Figure S5. ^{13}C-NMR of compound **9b**.

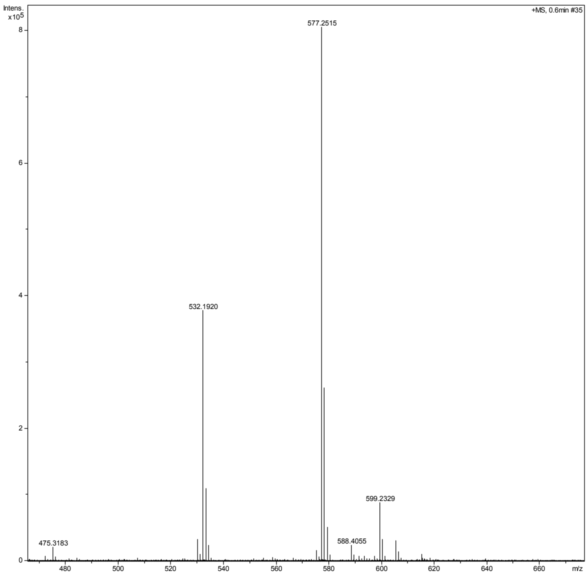

Figure S6. HR-ESI-MS spectra of compound **9b**.

4β-N-[(E)-(5-((Diethylamino)methyl)furan-2-yl)prop-2-en-1-amine]-4'-demethyl-4-desoxy-podophyllotoxin (**9c**)

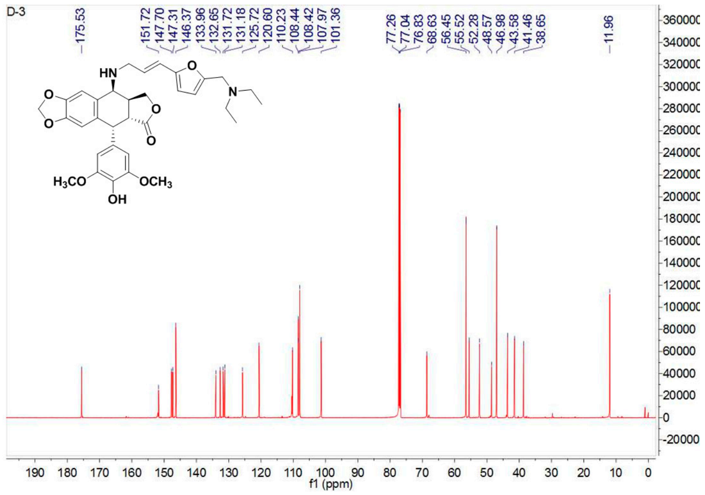

Figure S7. ^{1}H-NMR of compound **9c**.

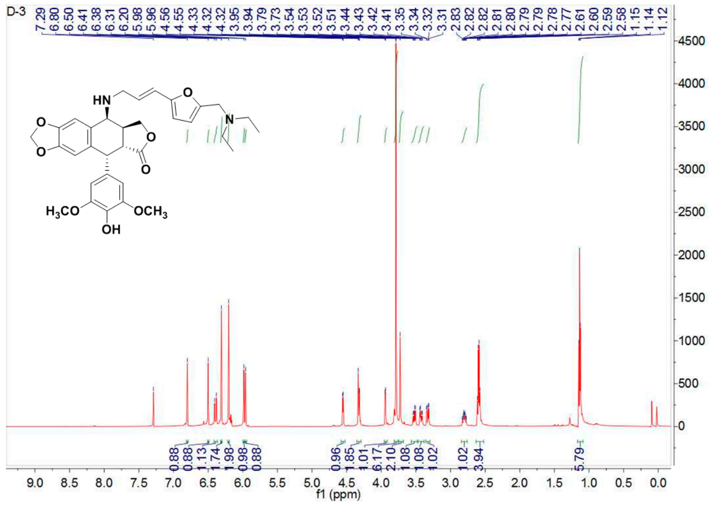

Figure S8. ^{13}C-NMR of compound **9c**.

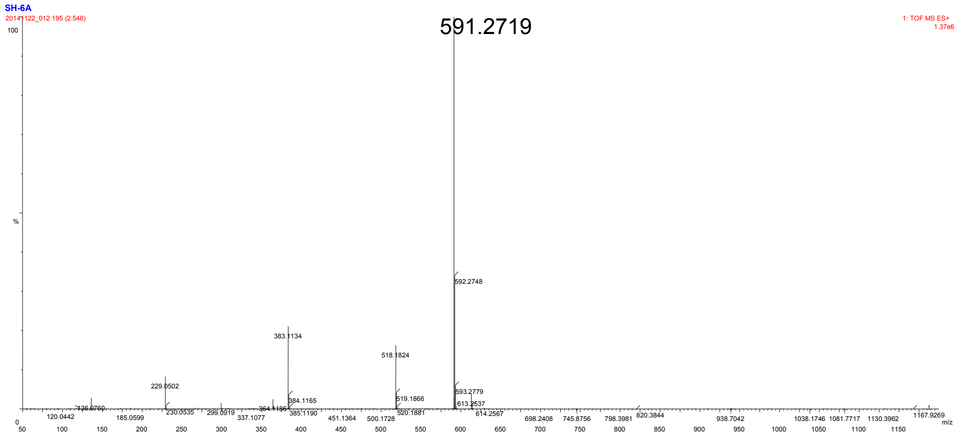

Figure S9. HR-ESI-MS spectra of compound **9c**.

4β-N-[(E)-(5-((Diethylamino)methyl)furan-2-yl)prop-2-en-1-amine]-4-desoxy-podophyllotoxin (**9d**)

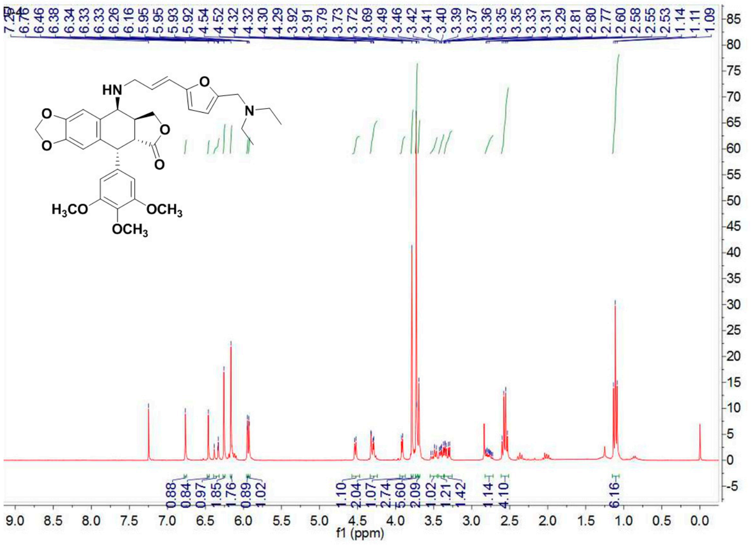

Figure S10. ^{1}H-NMR of compound **9d**.

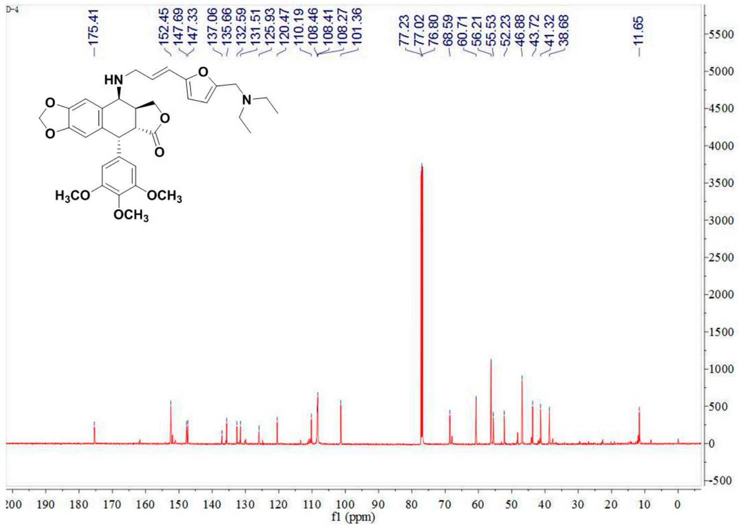

Figure S11. ^{13}C-NMR of compound **9d**.

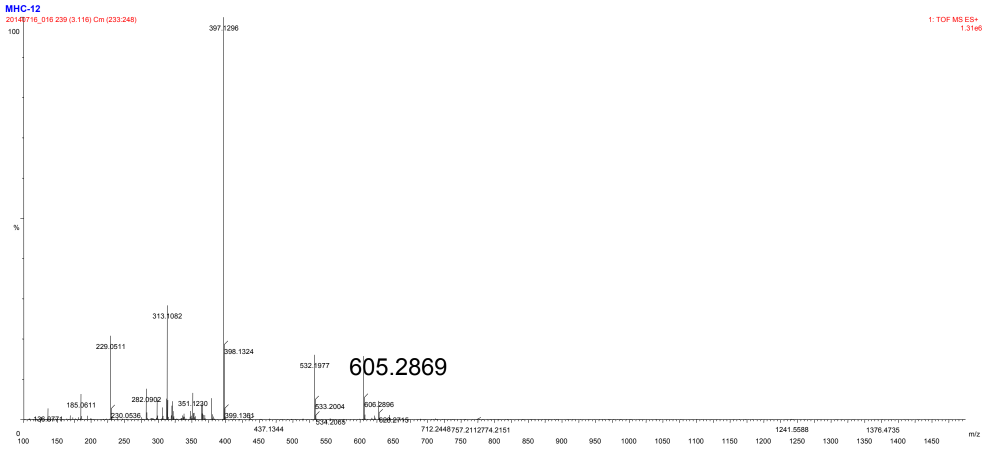

Figure S12. HR-ESI-MS spectra of compound **9d**.

4β-N-[(E)-(5-((4-Methylpiperazin-1-yl)methyl)furan-2-yl)prop-2-en-1-amine]-4′-demethyl-4-desoxy-podophyllotoxin (**9e**)

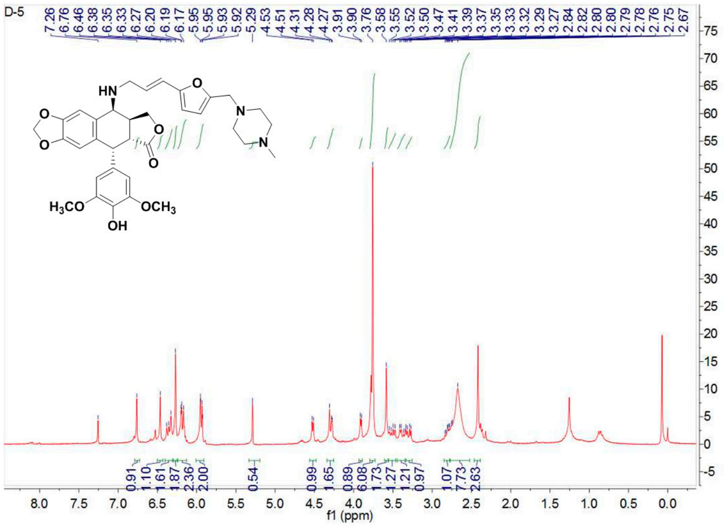

Figure S13. ^{1}H-NMR of compound **9e**.

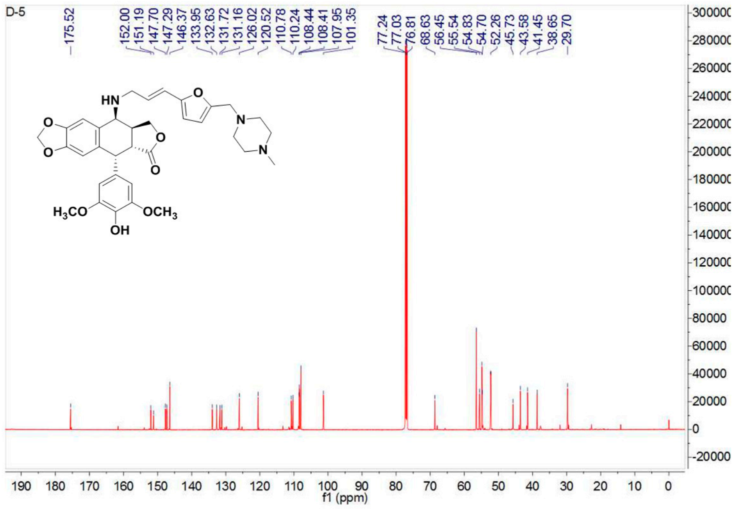

Figure S14. ^{13}C-NMR of compound **9e**.

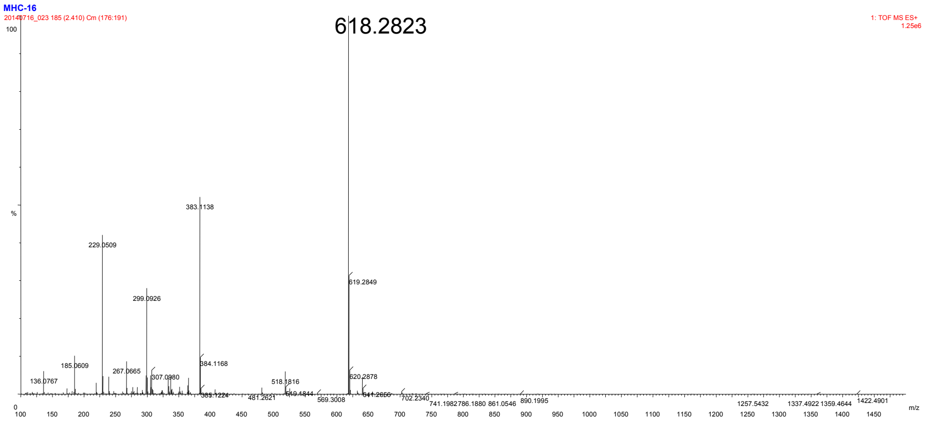

Figure S15. HR-ESI-MS spectra of compound **9e**.

4β-N-[(E)-(5-((4-Methylpiperazin-1-yl)methyl)furan-2-yl)prop-2-en-1-amine]l-4-desoxy-podophyllotoxin (**9f**)

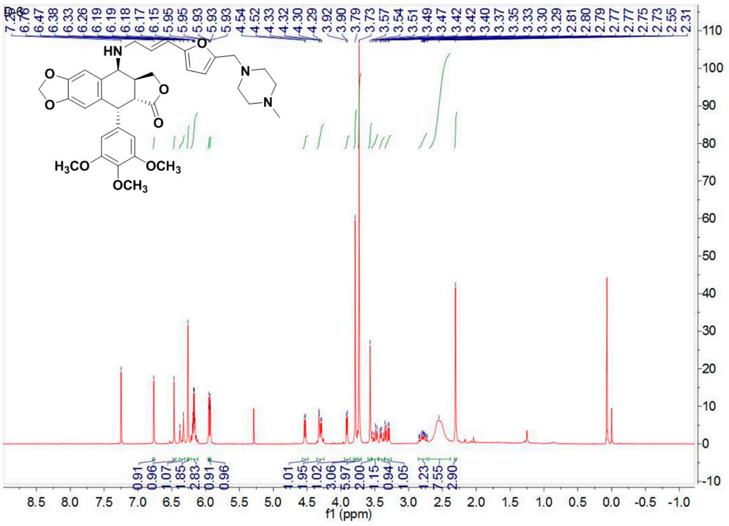

Figure S16. ^{1}H-NMR of compound **9f**.

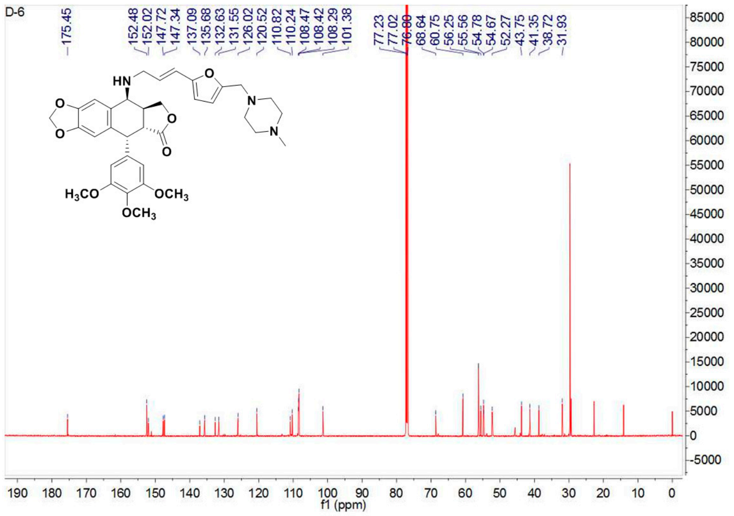

Figure S17. ^{13}C-NMR of compound **9f**.

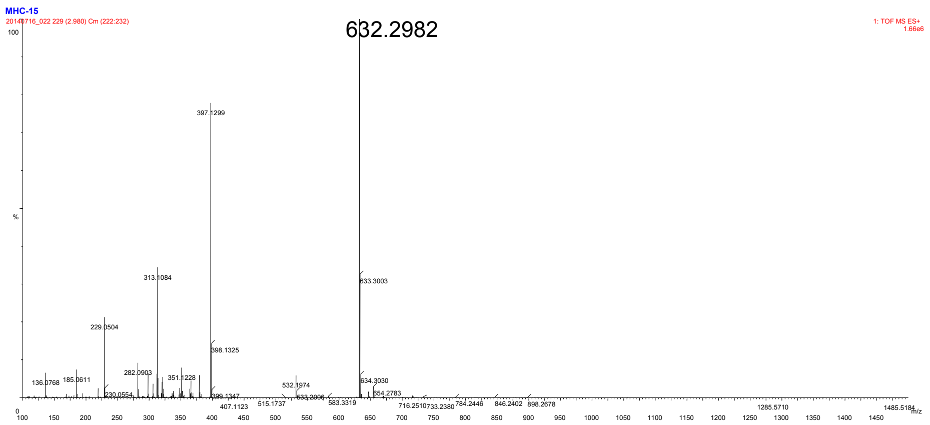

Figure S18. HR-ESI-MS spectra of compound **9f**.

4β-N-[(E)-(5-((4-Ethylpiperazin-1-yl)methyl)furan-2-yl)prop-2-en-1-amine]-4'-demethyl-4-desoxy-podophyllotoxin (**9g**)

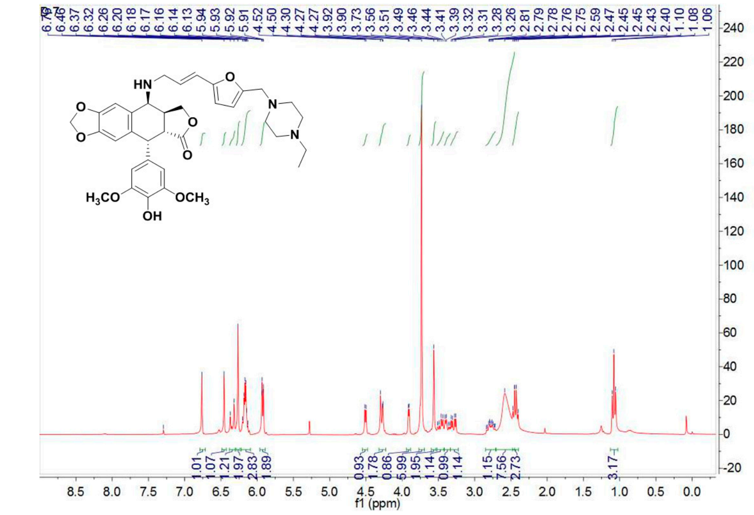

Figure S19. ^{1}H-NMR of compound **9g**.

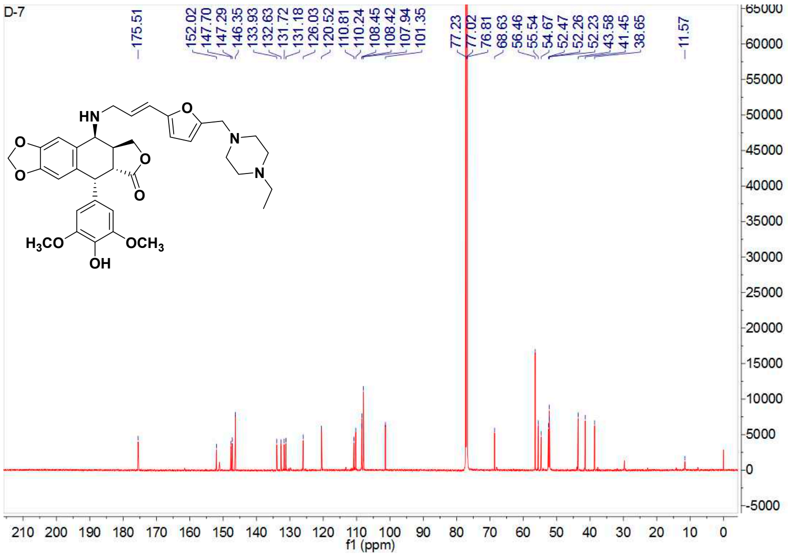

Figure S20. ^{13}C-NMR of compound **9g**.

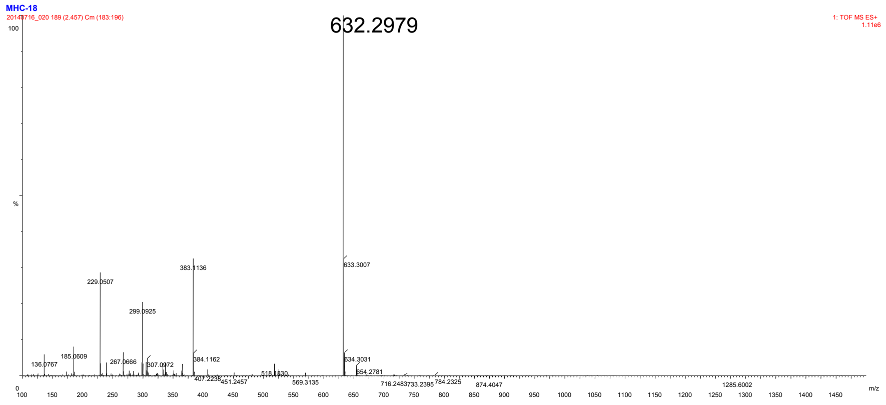

Figure S21. HR-ESI-MS spectra of compound **9g**.

4β-N-[(E)-(5-((4-Ethylpiperazin-1-yl)methyl)furan-2-yl)prop-2-en-1-amine]-4-desoxy-podophyllotoxin (**9h**)

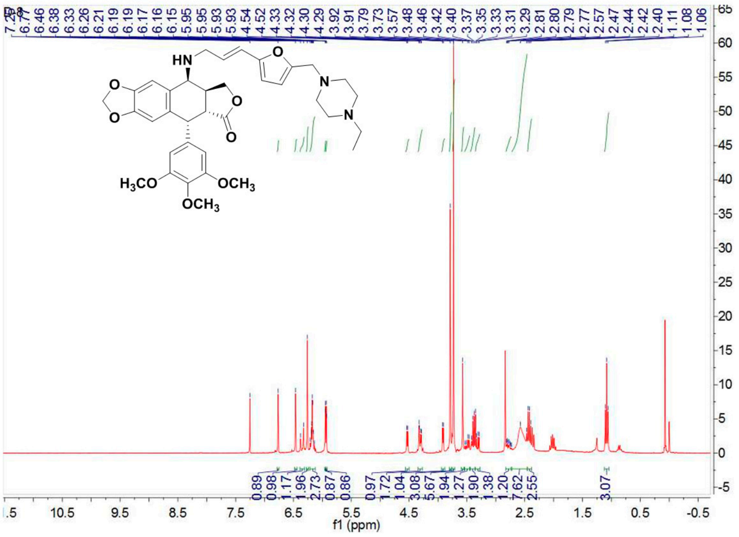

Figure S22. ^{1}H-NMR of compound **9h**.

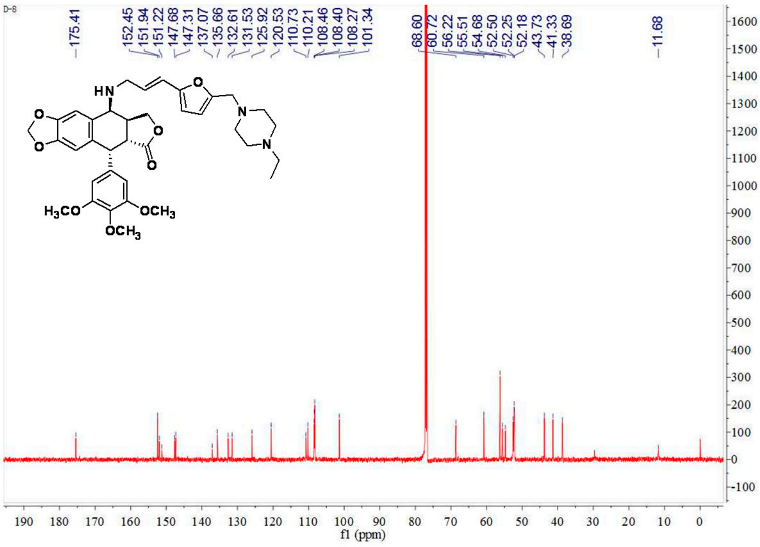

Figure S23. ^{13}C-NMR of compound **9h**.

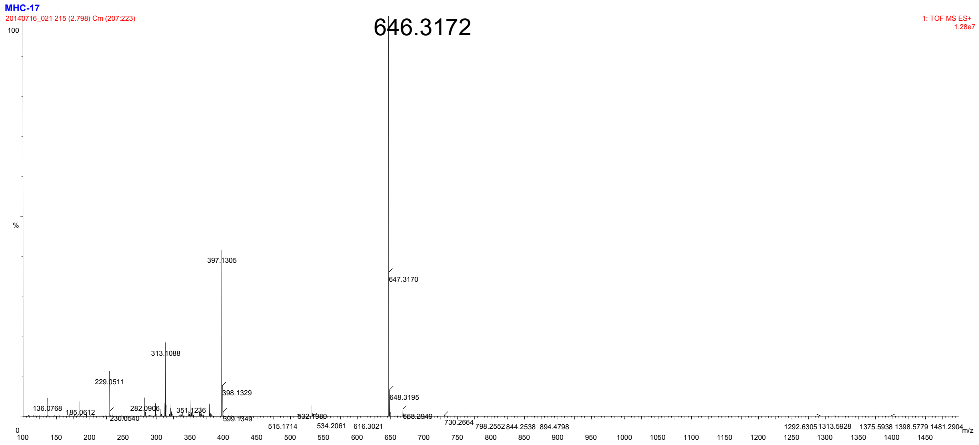

Figure S24. HR-ESI-MS spectra of compound **9h**.

4β-N-[(E)-(5-((4-(4-Fluorophenyl)piperazin-1-yl)methyl)furan-2-yl)prop-2-en-1-amine]-4'-demethyl-4-desoxy-podophyllotoxin (**9i**)

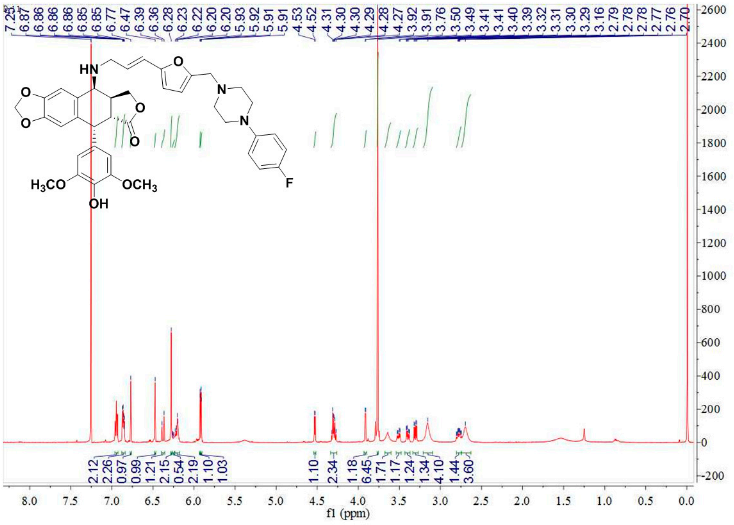

Figure S25. ^{1}H-NMR of compound **9i**.

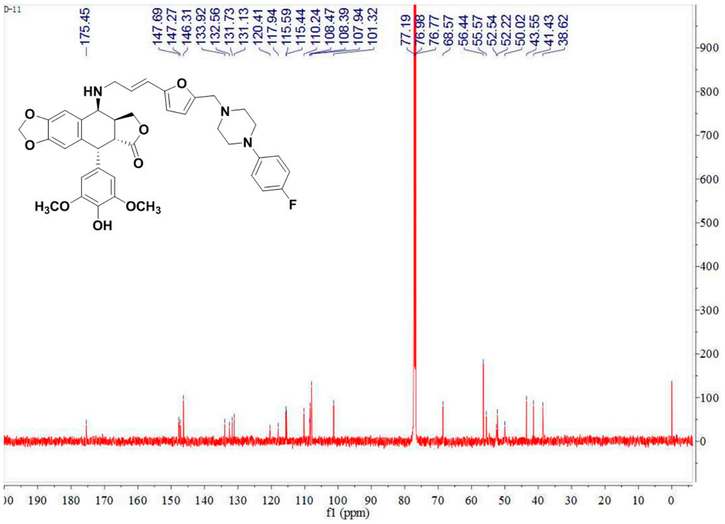

Figure S26. ^{13}C-NMR of compound **9i**.

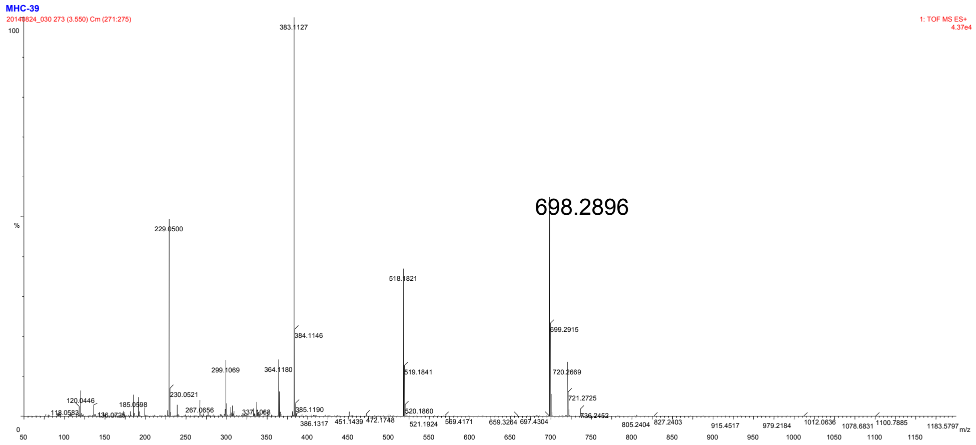

Figure S27. HR-ESI-MS spectra of compound **9i**.

4β-N-[(E)-(5-((4-(4-Fluorophenyl)piperazin-1-yl)methyl)furan-2-yl)prop-2-en-1-amine]-4-desoxy-podophyllotoxin (**9j**)

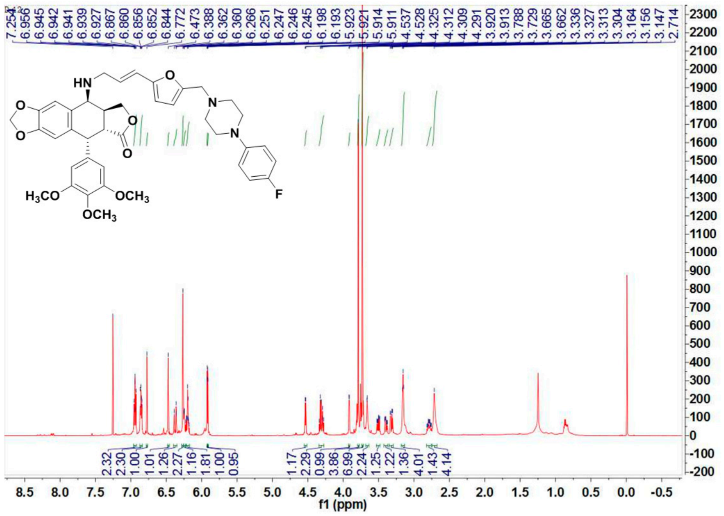

Figure S28. ^{1}H-NMR of compound **9j**.

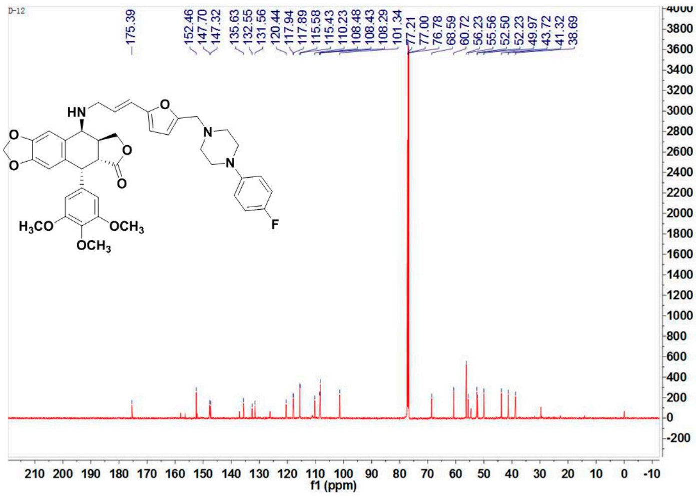

Figure S29. ^{13}C-NMR of compound **9j**.

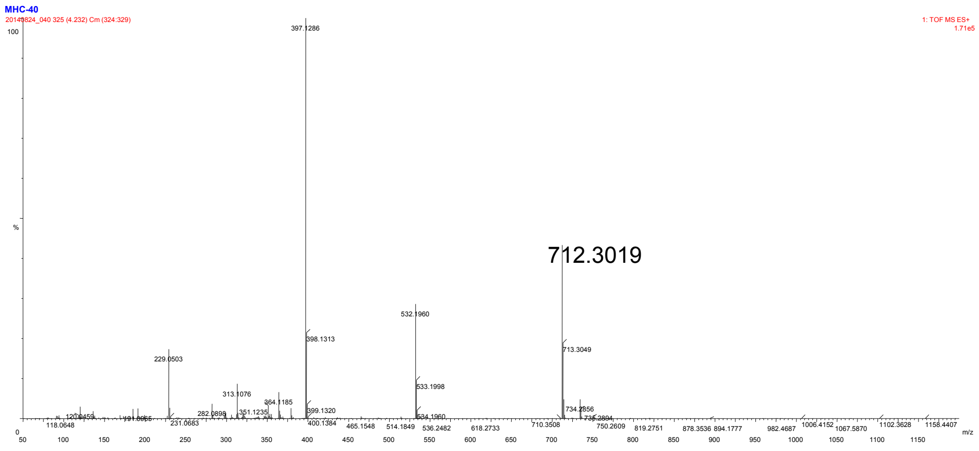

Figure S30. HR-ESI-MS spectra of compound **9j**.

4β-N-[*(E)*-(5-((4-(4-Nitrophenyl)piperazin-1-yl)methyl)furan-2-yl)prop-2-en-1-amine]-*4′-demethyl-4-desoxy-podophyllotoxin* (**9k**)

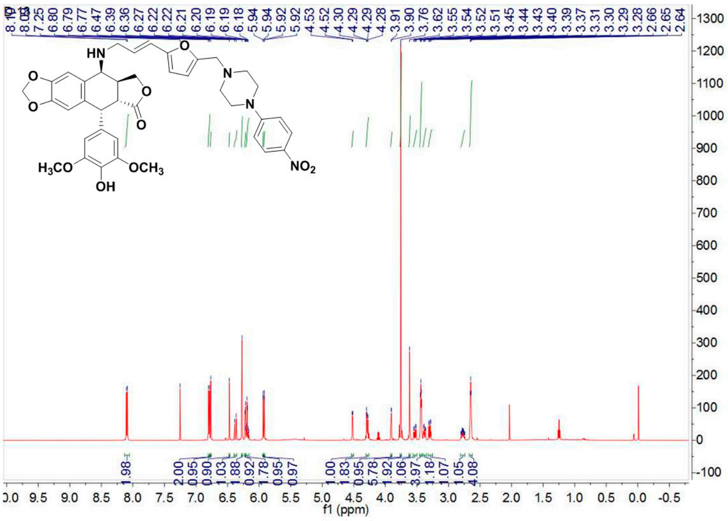

Figure S31. ^{1}H-NMR of compound **9k**.

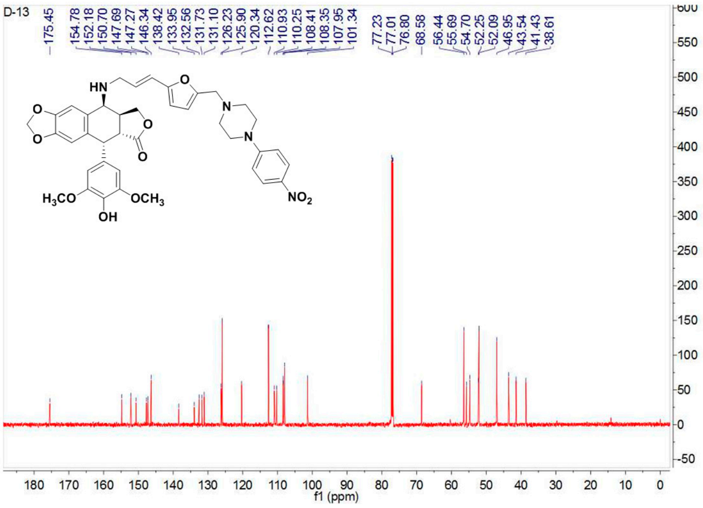

Figure S32. ^{13}C-NMR of compound **9k**.

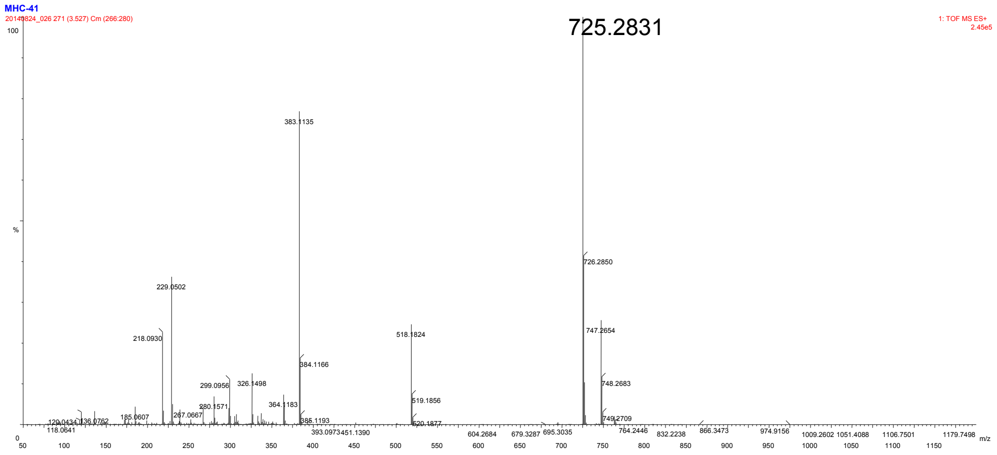

Figure S33. HR-ESI-MS spectra of compound **9k**.

4β-N-[(E)-(5-((4-(4-Nitrophenyl)piperazin-1-yl)methyl)furan-2-yl)prop-2-en-1-amine]-4-desoxy-podophyllotoxin (**9l**)

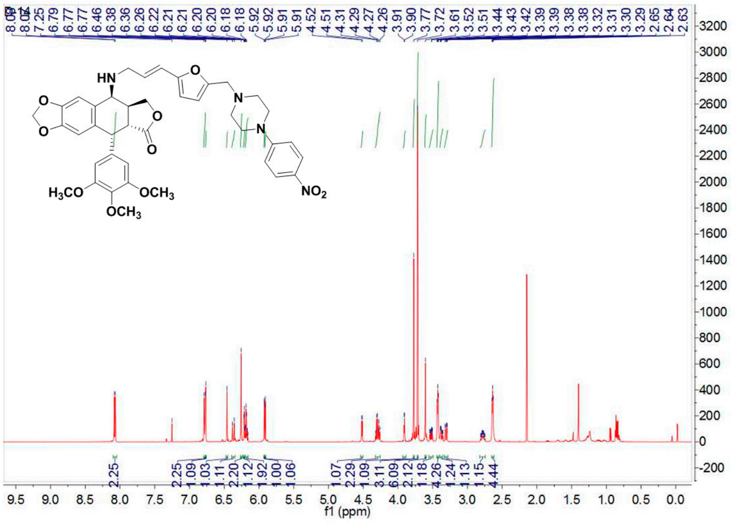

Figure S34. ^{1}H-NMR of compound **9l**.

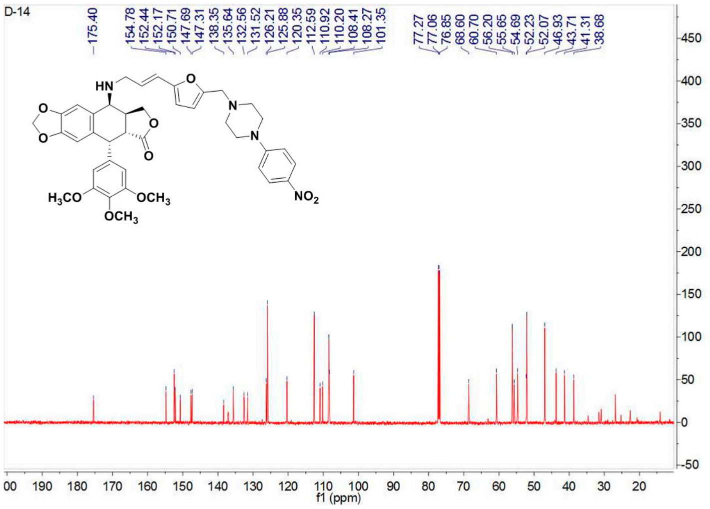

Figure S35. ^{13}C-NMR of compound **9l**.

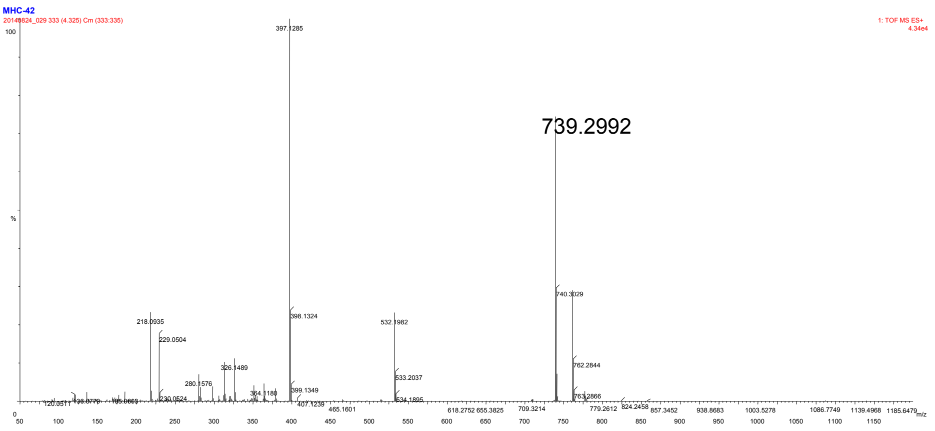

Figure S36. HR-ESI-MS spectra of compound **9l**.

4β-N-[(E)-(5-((4-Phenylpiperazin-1-yl)methyl)furan-2-yl)prop-2-en-1-amine]-4-desoxy-podophyllotoxin (**9m**)

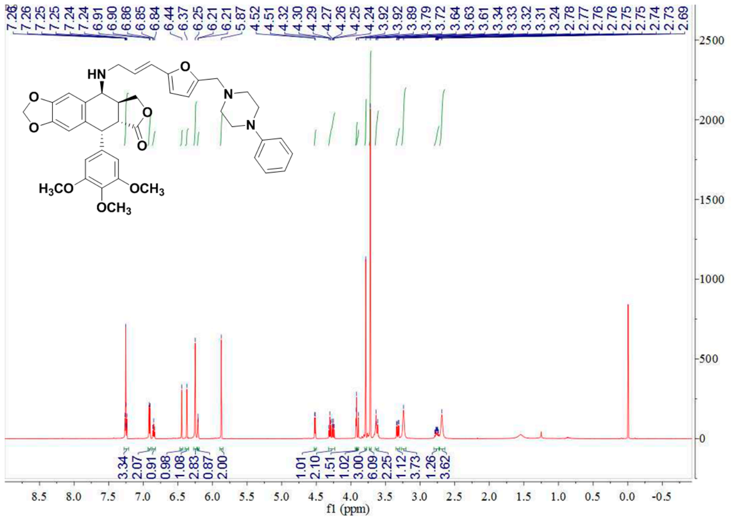

Figure S37. ^{1}H-NMR of compound **9m**.

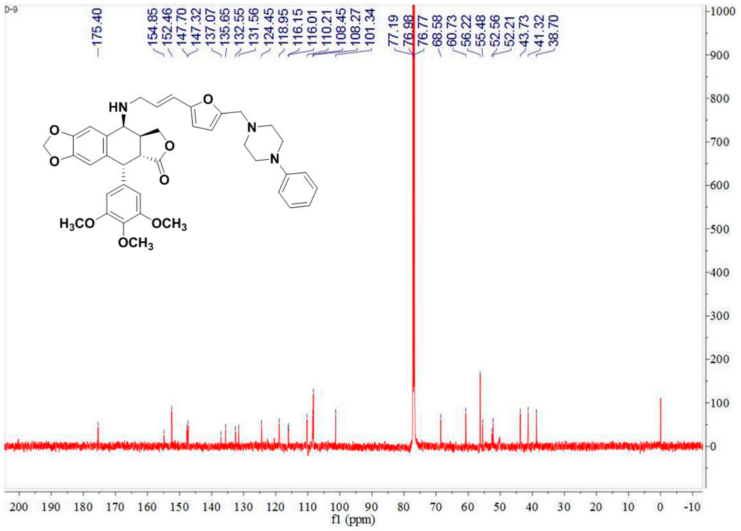

Figure S38. ^{13}C-NMR of compound **9m**.

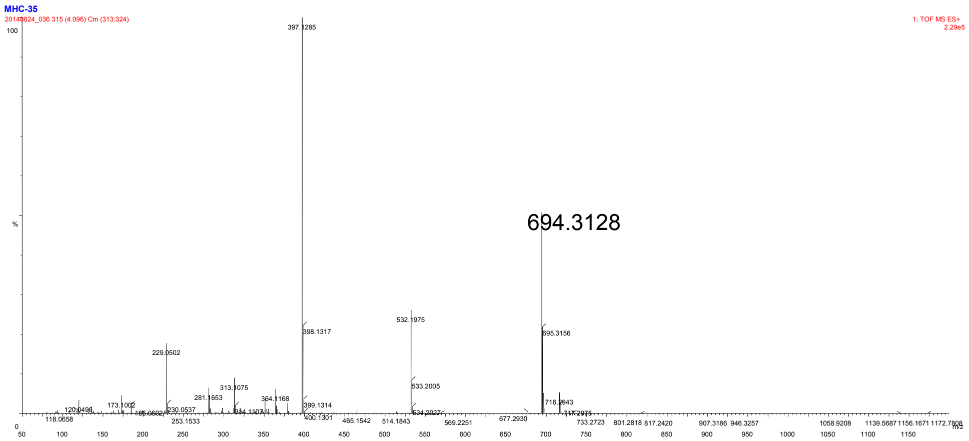

Figure S39. HR-ESI-MS spectra of compound **9m**.

4β-N-[(E)-(5-((4-(2-Fluorophenyl)piperazin-1-yl)methyl)furan-2-yl)prop-2-en-1-amine]-4-desoxy-podophyllotoxin (**9n**)

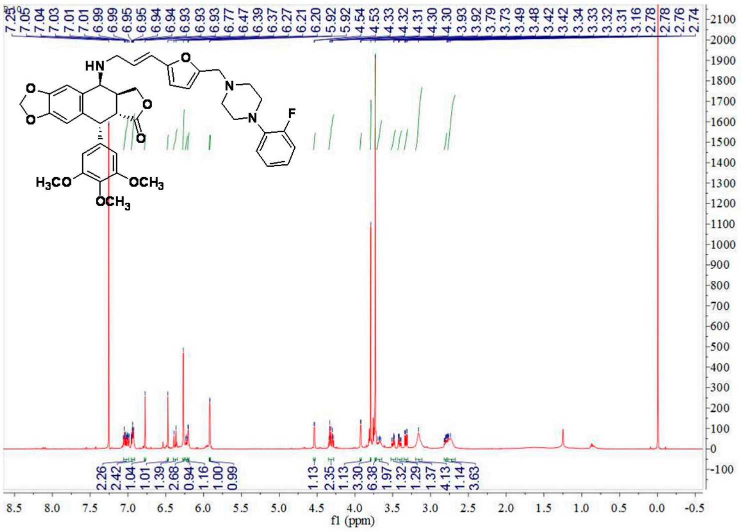

Figure S40. ^{1}H-NMR of compound **9n**.

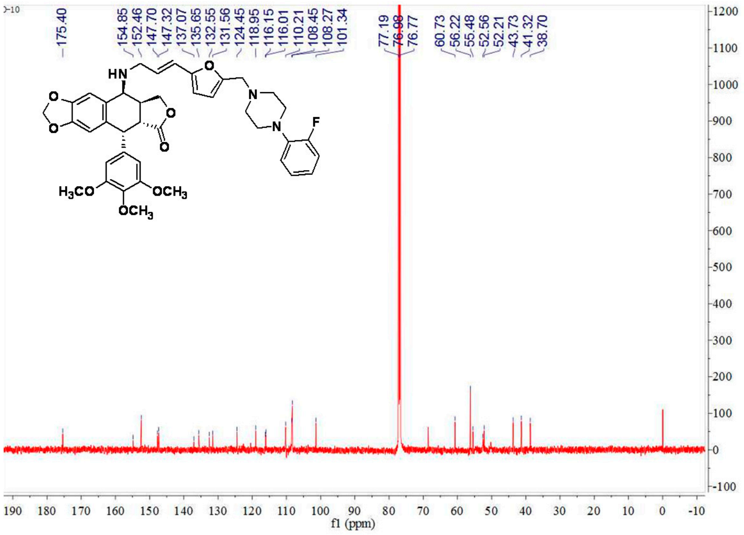

Figure S41. ^{13}C-NMR of compound **9n**.

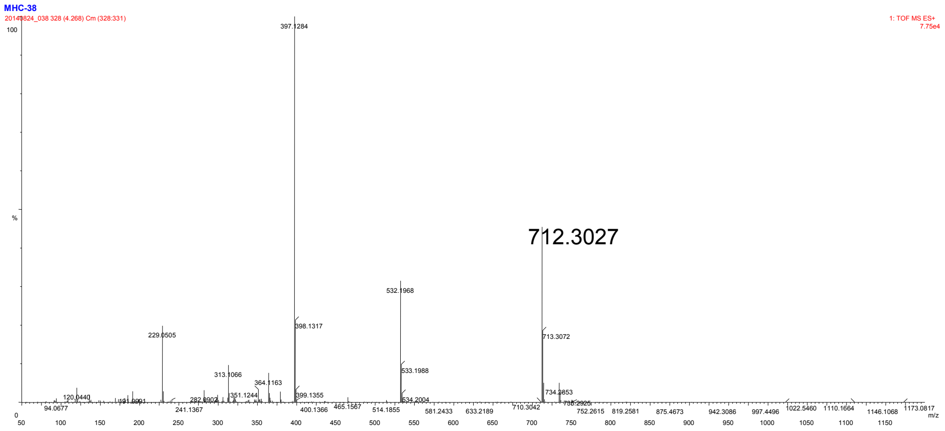

Figure S42. HR-ESI-MS spectra of compound **9n**.
